# Fundamental properties of the mammalian innate immune system revealed by multispecies comparison of type I interferon responses

**DOI:** 10.1371/journal.pbio.2004086

**Published:** 2017-12-18

**Authors:** Andrew E. Shaw, Joseph Hughes, Quan Gu, Abdelkader Behdenna, Joshua B. Singer, Tristan Dennis, Richard J. Orton, Mariana Varela, Robert J. Gifford, Sam J. Wilson, Massimo Palmarini

**Affiliations:** MRC-University of Glasgow Centre for Virus Research, Glasgow, United Kingdom; Fred Hutchinson Cancer Research Center, United States of America

## Abstract

The host innate immune response mediated by type I interferon (IFN) and the resulting up-regulation of hundreds of interferon-stimulated genes (ISGs) provide an immediate barrier to virus infection. Studies of the type I ‘interferome’ have mainly been carried out at a single species level, often lacking the power necessary to understand key evolutionary features of this pathway. Here, using a single experimental platform, we determined the properties of the interferomes of multiple vertebrate species and developed a webserver to mine the dataset. This approach revealed a conserved ‘core’ of 62 ISGs, including genes not previously associated with IFN, underscoring the ancestral functions associated with this antiviral host response. We show that gene expansion contributes to the evolution of the IFN system and that interferomes are shaped by lineage-specific pressures. Consequently, each mammal possesses a unique repertoire of ISGs, including genes common to all mammals and others unique to their specific species or phylogenetic lineages. An analysis of genes commonly down-regulated by IFN suggests that epigenetic regulation of transcription is a fundamental aspect of the IFN response. Our study provides a resource for the scientific community highlighting key paradigms of the type I IFN response.

## Author summary

The type I interferon (IFN) response is triggered upon sensing of an incoming pathogen in an infected cell and results in the expression of hundreds of IFN-stimulated genes (ISGs, collectively referred to as ‘the interferome’). Studies on the interferome have been carried out mainly in human cells and therefore often lack the power to understand comparative evolutionary aspects of this critical pathway. In this study, we characterized the interferome in several animal species (including humans) using a single experimental framework. This approach allowed us to identify fundamental properties of the innate immune system. In particular, we revealed 62 ‘core’ ISGs, up-regulated in response to IFN in all vertebrates, highlighting the ancestral functions of the IFN system. In addition, we show that many genes repressed by the IFN response normally function as regulators of cell transcription. ISGs shared by multiple species have a higher propensity than other genes to exist as multiple copies in the genome. Importantly, we observed that genes have arisen as ISGs throughout evolution. Hence, every animal species possesses a unique repertoire of ISGs that includes core and lineage-specific genes. Collectively, our data provide a framework on which it will be possible to test the role of the IFN response in pathogen emergence and cross-species transmission.

## Introduction

Most emerging human viruses have an animal origin [1]. The increase in the global human population, international travel, and ecological changes, in addition to changes in agricultural practices, has led to complicated interactions between wildlife, domestic species, and humans that has enhanced the opportunities for cross-species transmission of known, as well as newly discovered, viruses [1,2]. Physical and molecular components of the innate immune system represent early barriers to incoming viruses that must be overcome in order for an infection to prevail. In vertebrates, one of the key innate immune defences against virus infection is the interferon (IFN) system. Type I interferons (including IFN-β and IFN-α among others; here referred to simply as IFN), type II interferons (IFN-γ), and type III interferons (IFN-λ) are cytokines with antipathogen, immunomodulatory, and proinflammatory properties. The IFN system is usually stimulated by the detection of pathogen signatures, known as pathogen-associated molecular patterns (PAMPs), resulting in the secretion of IFN. In turn, IFN signalling results in the up-regulation of hundreds of interferon-stimulated genes (ISGs), collectively referred to as the type I ‘interferome’ (here simply the ‘interferome’) [3,4]. Unsurprisingly, given the importance of IFN in combatting pathogen invasion, there are numerous examples of coevolutionary arms races between ISGs and invading pathogens [5,6]. However, previous studies investigating ISG transcription have focused on the interferomes of single species [7,8]. Despite resources such as the Interferome database [9], variations in experimental and bioinformatic approaches make comparing interferomes derived from divergent species and collected from different studies a difficult prospect if significant technical caveats and confounding factors are to be avoided. Here, we used the same RNA sequencing (RNAseq) approach on 10 animal species to deliver a snapshot of the genes that are differentially expressed in cells (fibroblasts) in a type I IFN-induced antiviral state. This snapshot of the interferome from a single cell type at one point in time cannot capture the entire temporal and tissue-specific complexity of the interferome. Nonetheless, using this comparative approach, we have uncovered fundamental paradigms of the IFN system.

## Results

### Interferomes differ among evolutionary lineages

We first determined the individual interferomes of *Homo sapiens* (human), *Rattus norvegicus* (rat), *Bos taurus* (cow), *Ovis aries* (sheep), *Sus scrofa* (pig), *Equus caballas* (horse), *Canis lupus familiaris* (dog), *Myotis lucifugus* (little brown bat, microbat), *Pteropus vampyrus* (large flying fox, fruit bat), and *Gallus gallus* (chicken) cells. Interferomes were obtained from cells stimulated with type I IFN and experimentally confirmed as being in an antiviral state as described in the Materials and methods (S1 Fig). In our study, we defined an ISG as a gene up-regulated by IFN with a false discovery rate (FDR) of <0.05, regardless of the extent to which it was up-regulated. To facilitate mining of the data, we also developed an open access webserver (http://isg.data.cvr.ac.uk) capable of filtering the dataset based upon user-defined criteria.

**Fig 1:**
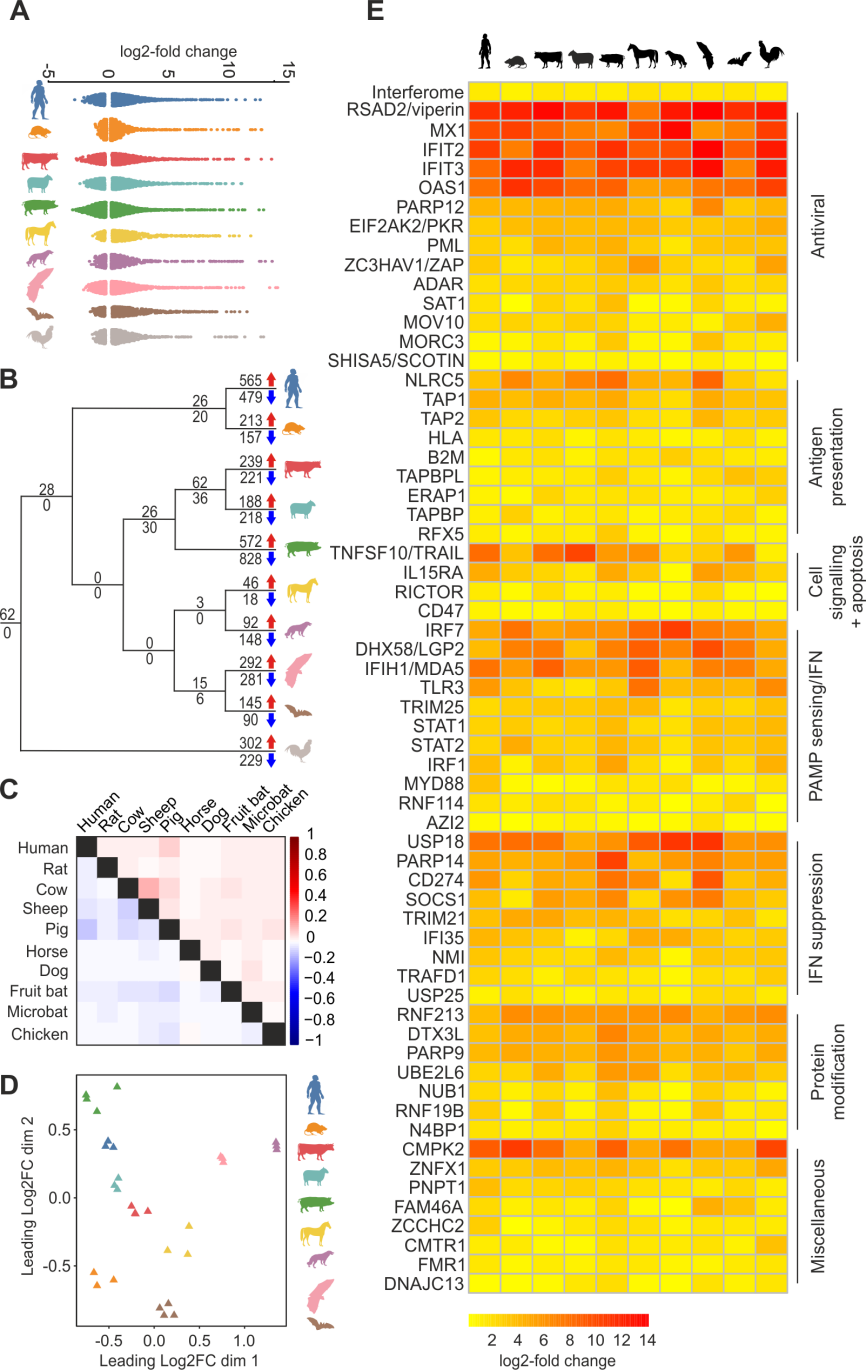
Patterns of differential gene expression in response to type I IFN among cells from the 10 vertebrate species used in this study. (A) The patterns of differential expression of ISGs and IRGs are broadly similar in cells derived from 10 different animal species. Each dot in the panel represents a gene that is differentially expressed in response to type I IFN treatment (S1 Data). (B) Number of ISGs (above line, red arrow) and IRGs (below line, blue arrow) are plotted on the branches of a simplified phylogenetic tree (branch lengths are not shown to scale). ISGs common to every species (*n* = 62) are located at the root of the tree with an additional 28 ISGs up-regulated by all mammalian species used in this study. At the tips of the tree lie genes that are only up- or down-regulated in an individual species in our study. (C) Normalised correlation matrix showing pairwise comparisons between ISGs (red) and IRGs (blue) of the indicated animal species (S1 Data). (D) A PCA of the log2FC data of the one-to-one orthologs up-regulated by all nine mammalian species used in this study (S1 Data). Each point represents an animal or experiment, coloured according to species. The distribution of the samples reflects expression patterns. Separate animals/experiments cluster according to species, with the pig and human showing similar patterns. (E) A heatmap of the relative expression of the 62 vertebrate core ISGs. The first row (labelled as ‘Interferome’) represents the average log2FC of all up-regulated ISGs for each animal species. Up-regulated paralogs have been averaged in the case of genes for which there are expansions; for example, the rat has two copies of MX1 compared to the single copy in the remaining species (S1 Data). IFN, interferon; IRGs, interferon-repressed genes; ISGs, interferon-stimulated genes; log2FC, log2 fold change; PCA, principal component analysis.

The absolute number of ISGs differentially expressed in each species varied (S1 Table), but their pattern of differential expression in response to type I IFN was remarkably similar (Fig 1A). The presence of shared ISGs at specific nodes on a schematic phylogeny provided evidence that interferomes have been sculpted over time by lineage-specific pressures possibly exerted by different pathogens (Fig 1B). As expected, we observed that the most closely related species in our dataset, cows and sheep, showed the greatest similarity in the genes they up-regulate (Fig 1C). However, we also observed substantial levels of similarity in the interferomes of some species that are more distantly related phylogenetically, most notably pigs and humans (Fig 1C). Interestingly, this finding was reflected in a principal component analysis of the 35 one-to-one (i.e., single copy) ISG orthologs up-regulated by every mammalian species in our study (see below), whereby the patterns of differential expression were again similar between humans and pigs (Fig 1D).

Every species possessed unique ISGs that were not up-regulated by IFN in any of the other nine species. Furthermore, certain ISGs present in our dataset (despite being up-regulated by IFN >2 log2 fold change [log2FC]) had few (if any) orthologs in the other genomes in the Ensembl database. Examples include a gene (RGD1561157) that is annotated on chromosome 10 of the rat genome and two chicken genes (Ensembl IDs ENSGALG00000019325 and ENSGALG00000020899).

### Core ISGs

We identified a core set of 62 genes (“core vertebrate ISGs”, hereinafter core^vert^ ISGs) that were up-regulated by IFN by all 10 species analysed in this study, with an additional 28 genes up-regulated specifically in the nine mammalian species (“core mammalian ISGs”, hereinafter core^mamm^ ISGs) (Table 1). The core^vert^ ISGs represent the ancestral functions of the IFN system and include genes encoding proteins broadly involved in (i) orchestrating antigen presentation, (ii) IFN induction and response, (iii) IFN suppression, (iv) ubiquitination and protein degradation, (v) cell signalling and apoptosis, and (vi) antiviral responses (Table 1, Fig 1E).

**Table 1:** Core ISGs.

Antigen presentation	Antiviral	Ubiquitin + protein modification	PAMP sensing + IFN pathway	IFN suppression	Cell signalling and apoptosis	Miscellaneous
B2M	ADAR	DTX3L	AZI2	CD274	CASP8*	C2*
ERAP1	APOL1,2,3,4*	HERC6*	cGAS (MB21D1)*	IFI35	CD47	CMPK2
HLA^#^	C19orf66 (IRAV)*	N4BP1	IRF1	NMI	IL15RA	CMTR1
NLRC5	IFIT2	NUB1	IRF7	PARP14	LGALS9*	DNAJA1*
PSMA5*	IFIT3	PARP9	IRF9*	SOCS1	RICTOR	DNAJC13
PSMB8*	ISG15*	RBCK1*	LGP2 (DHX58)	TRAFD1	TRAIL (TNFSF10)	EHD4*
PSMB9*	ISG20*	RNF19B	MDA5 (IFIH1)	TRIM21		FAM46A
PSMB10*	MORC3	RNF213	MYD88	USP18		FMR1
PSME1*	MOV10	RNF31*	RIG-I (DDX58)*	USP25		PNPT1
PSME2*	MX1	UBA7*	RNF114			SERTAD1*
RFX5	OAS1	UBE2L6	STAT1			SLC25A28*
TAP1	PARP12		STAT2			SP110*
TAP2	PKR (EIF2AK2)		TLR3			TDRD7*
TAPBP	PML		TRIM25			WARS*
TAPBPL	RSAD2 (viperin)					XAF1*
	SAT1					ZCCHC2
	SCOTIN (SHISA5)					ZNFX1
	ZAP (ZC3HAV1)					

* = ISGs shared only among the mammalian species analysed in this study (core^mamm^ ISGs)

^#^ = Includes HLA-A, -B, -C, -E, -F, -G

**Abbreviations:** IFN, interferon; ISGs, interferon-stimulated genes; PAMP, pathogen-associated molecular pattern

Nine of the 62 core^vert^ ISGs (e.g., various HLA genes, TAP1, ERAP1, etc.) are involved in the generation, trimming, loading, and presentation of MHC-I–restricted antigens, thus providing a direct link between the IFN response and the adaptive immune response via the CD8 T-cell response (Table 1). Additionally, components of the immunoproteasome (e.g., PSMB8 and PSMB9) were specifically up-regulated in the mammalian core (Table 1), reflecting previous studies reporting the absence of the immunoproteasome in birds [10].

**Fig 2:**
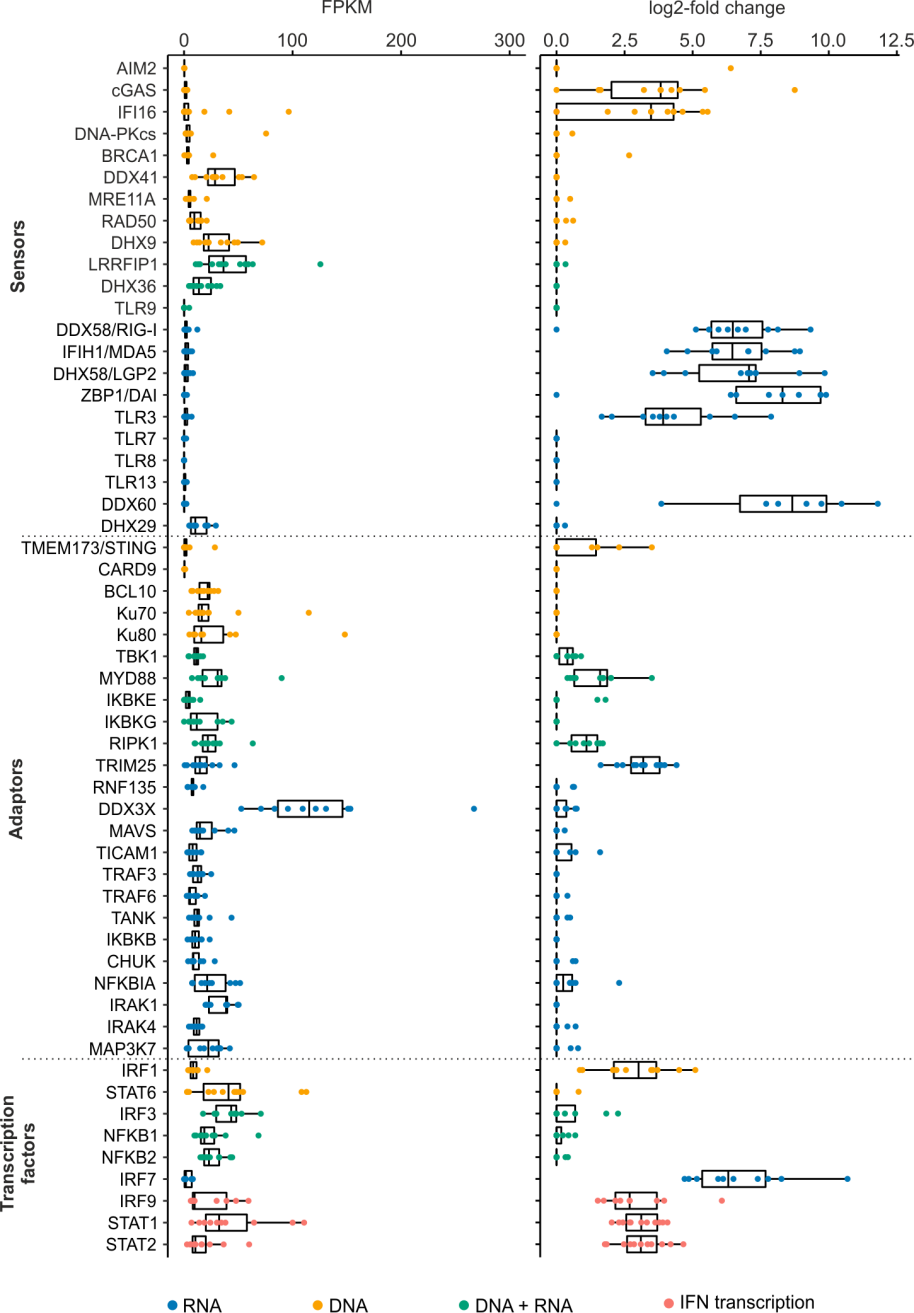
Basal transcription levels and IFN-induced expression of genes related to PAMP sensing and IFN induction and response. Boxplots showing differential expression (log2FC) in response to IFN and basal transcription levels (expressed as FPKM) of genes associated with pattern recognition (sensors), downstream signal transduction (adapters), and transcription factors related to either IFN induction or response (transcription factors). Every ortholog for each gene is indicated with a dot coloured according to their presence in the DNA-, RNA-, or both DNA- and RNA-sensing pathways (S1 Data). FPKM, fragments per kilobase mapped values; IFN, interferon; log2FC, log2 fold change; PAMP, pathogen-associated molecular pattern.

We found that various core^vert^ ISGs are involved in IFN induction and response, including pattern recognition receptors, the key adaptor molecule MyD88, and transcription factors. The classical sensors for RNA PAMPs, including IFIH1/MDA5, DHX58/LGP2, and TLR3 (and RIG-I/DDX58 in the mammals) were among the core^vert^ ISGs (Table 1, Fig 2). RIG-I is known to be absent in chickens (and other galliformes) but is present and active in other birds, including ducks and geese [11–13]. Furthermore, TRIM25, a ubiquitin E3 ligase responsible for ubiquitinating RIG-I, was also a core^vert^ ISG [14]. DAI/ZBP1, which was originally classified as a DNA sensor but is now thought to be an RNA sensor [15], was also highly up-regulated by IFN in every mammalian species except humans (Fig 2). No DNA sensors were found among the core^vert^ ISGs. However, cGAS was found to be an ISG in every mammalian species in this study (Fig 2). By contrast, the DNA sensor AIM2 was only up-regulated in human cells. Interestingly, whilst the basal expression (defined in terms of fragments per kilobase mapped values [FPKM] in the absence of IFN treatment) of RNA sensors was very low, many of the genes in the literature associated with DNA sensing are constitutively transcribed (Fig 2).

With the exception of MyD88, a key adaptor involved in both the RNA- and DNA-sensing pathways [16], we observed limited up-regulation among genes involved downstream of nucleic acid detection (Fig 2). On the other hand, core ISGs included key transcription factors involved in IFN induction and response (IRF1, IRF7, STAT1 and STAT2 are all core^vert^ ISGs in addition to IRF9 among the core^mamm^ ISGs) (Fig 2). Importantly, several ISGs that play a role in the suppression of the IFN system, including USP18, USP25, IFI35, and SOCS1 were up-regulated in all species under examination. The encoded proteins of these genes target different points in the IFN response. Thus, negative regulation of the IFN response is multifaceted and a fundamental, ancestral failsafe necessary to avoid excessive/perpetual up-regulation of IFN-induced pathways. Among the core^vert^ ISGs, we found several genes relating to ubiquitination, such as the ring finger proteins RNF213 and RNF19B (Table 1, Fig 1E), highlighting protein modification as part of the IFN response. Interestingly, N4BP1, originally identified as a target of Nedd4-mediated ubiquitination, has not previously been directly linked to the IFN response.

**Fig 3:**
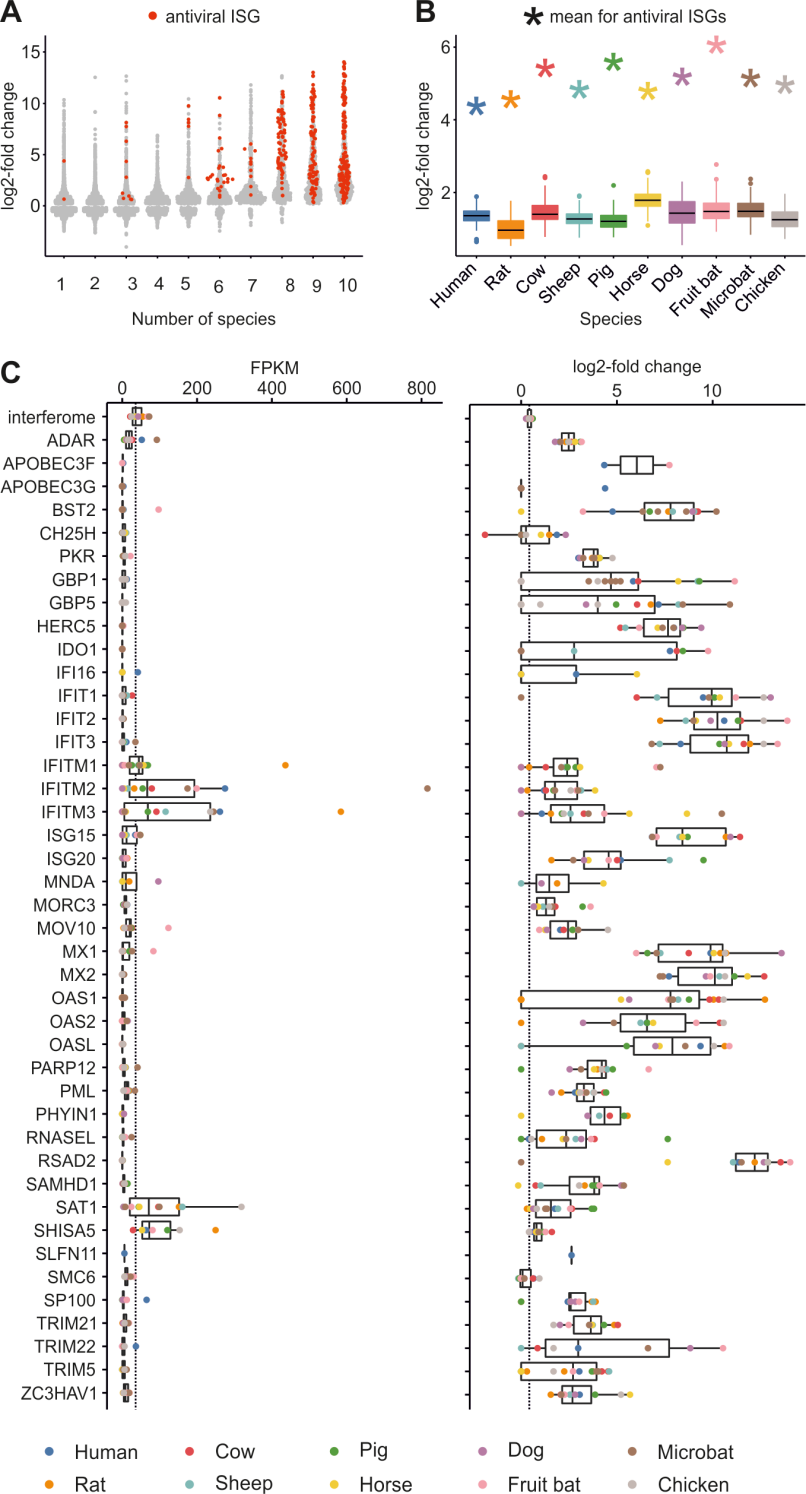
Properties of antiviral ISGs. (A) Sinaplot showing the differential expression values (log2FC) of 40 genes previously published as exerting antiviral activity (red dots) (S2 Table) as opposed to the rest of the ISGs (grey dots). ISGs (including antiviral ISGs) were allocated to bins according to the number of species in which they were found to be up- or down-regulated (i.e., the core^vert^ ISGs are bin 10). The majority of antiviral ISGs (red) were found to be up-regulated in at least eight species (S1 Data). (B) Graph showing the extent of up-regulation of antiviral ISGs compared to nonantiviral ISGs. The mean log2FC of 40 known antiviral ISGs (indicated with an asterisk on the plot) is compared to 100 samplings of 40 randomly selected ISGs from the interferome of each species (box and whiskers). In all cases, antiviral ISGs are up-regulated to a significantly greater extent as compared to nonantiviral ISGs (*P* < 0.01 for each species). The code used for random sampling and the generation of Fig 3B is available in S2 Data, with the required input files available as S5 Data and S6 Data. (C) Boxplots showing basal transcription levels (expressed as FPKM) and differential expression (log2FC) in response to IFN of known antiviral ISGs as in panels A and B. Every ortholog for each gene is indicated with a dot coloured according to species. The median FPKM value for the entire interferome is indicated with a broken line (S1 Data). FPKM, fragments per kilobase mapped values; IFN, interferon; ISGs, interferon-stimulated genes; log2FC, log2 fold change.

The core^vert^ ISGs contained 14 IFN-induced antiviral factors such as MX1, IFIT2, and viperin (Table 1). Interestingly, when we assembled a list of 40 genes that either create a cellular environment hostile to or act directly upon the virus lifecycle (based upon the scientific literature; S2 Table), we noticed that 75% of these antiviral genes were ISGs in at least eight of the 10 species analysed in this study (Fig 3A). In addition, antiviral ISGs were up-regulated to a significantly higher extent than randomly sampled ISGs (*P* < 0.01 for each species, Fig 3B). Furthermore, some well-studied antiviral ISGs were not up-regulated by IFN in certain species. For example, OAS2 was not up-regulated in the rat, SAMHD1 was not up-regulated in the horse, OASL was not up-regulated in either the cow or the sheep, and the IFITM genes were not up-regulated in the dog (Fig 3C). In general, genes encoding antiviral factors were transcribed to minimal levels in the absence of IFN. Indeed, the median FPKM level for antiviral genes was lower than that of the overall interferome for every antiviral factor except SAT1, SHISA5, and the IFITM genes. Of interest, we noticed particularly high FPKM values for IFITM1 and 3 in the rat and IFITM2 in the microbat (Fig 3C).

Two core^vert^ genes, CD47 and IL15RA, encode proteins involved in signalling to components of the adaptive immune system. CD47 is involved in a variety of biological roles, including leukocyte and dendritic cell migration, the development of antigen presenting cells, and immune apoptosis, and it also provides a ‘don’t eat me’ signal [17]. The IL15–IL15Rα axis is well characterised as being important in the promotion of both natural killer cells and a variety of T cell populations, including activated CD8 T cells [18].

### Genes previously unrelated to the IFN response

Among the core^vert^ ISGs, we identified a number of genes with few or no reported associations with the type I IFN response (Table 1). Several of these genes have been studied extensively, but not always in the context of the IFN response. DNAJC13, for example, is reported to be involved in endosome trafficking, and it has been closely linked to Parkinson’s disease [19]. Zinc Finger CCHC-Type Containing 2 (ZCCHC2) has nucleic acid–binding properties and, interestingly, contains a single nucleotide polymorphism (SNP) associated with insect bite hypersensitivity in Exmoor ponies [20]. The fragile X mental retardation (FMR1) gene encodes an RNA-binding protein that plays a role in intracellular RNA transport and in the regulation of translation of target mRNAs. FMR1 was not previously linked to the IFN response, although it has recently been shown to be a proviral factor for influenza virus and previously was shown to induce mild restriction of HIV-1 [21,22]. Cap methyltransferase 1 (CMTR1), also known as ISG95, binds to RNA pol II and is a 2′-O-ribose methyltransferase that participates in the conversion of cap0 to cap1 type transcripts [23]. Interestingly CMTR1 is also known as an important component of IFIT-mediated antiviral activity [24]. We hypothesise that these genes, as core^vert^ ISGs, likely play fundamental roles in host immunity that are underappreciated or have yet to be fully determined.

Our data also indicate a relatively underappreciated link between local synthesis of early components of the complement system and the type I IFN response [25,26]. C2 was among our core^mamm^ ISGs. In addition, we found that C1r and C1s, essential components of the C1 complex, were up-regulated by IFN in cells from all species analysed in our study with the exception of the cow. Interestingly, this was also the case for a negative regulator of C1r and C1s (SERPING1/C1-INH).

### Interferon-repressed genes and epigenetic controls of the IFN response

Our data enabled an unprecedented opportunity to investigate the interferon-repressed genes (IRGs), which, to date, have received comparatively little attention with regards to their involvement in the innate immune system. Unlike ISGs, the extent of down-regulation of IRGs across all the 10 species used in this study was relatively modest (overall average −0.56 log2FC for IRGs as compared to 1.64 log2FC for ISGs). We found no IRGs shared by all species, although this may reflect the low fold change in expression and/or greater variability in the response of individual genes. This result could also imply that there has been less conservation of the down-regulated genes over time. The most consistently down-regulated genes were FAM117B and KDM5B, which were both significantly down-regulated in eight of the 10 species analysed in this study. Relatively little is known about the function of FAM117B with the exception that it is a risk factor for sarcoidosis [27]. On the other hand, it has been shown that suppression of the KDM5B gene product, a H3K4 demethylase causing transcriptional repression, results in increased expression of IFN-β and other inflammatory cytokines following infection with respiratory syncytial virus (RSV) [28]. We also noticed that, with the exception of the rat, every species analysed down-regulated at least one KDM gene in response to IFN.

It is already established that another form of epigenetic control, acetylation, is also required for robust ISG transcription [29]. ANP32A, a protein involved in acetyltransferase inhibition [30], was down-regulated in five species. Interestingly, ANP32A has been shown to be a host component necessary for influenza virus replication and influences the ability of the virus to replicate in a given animal species [31].

Our data have thus revealed that key epigenetic factors regulating ISG transcription are themselves frequently responsive to IFN treatment. Along these lines, we also investigated the presence of noncoding RNAs (ncRNAs), a class of RNA molecule increasingly recognised as being important in the antiviral response [32,33]. We found that in human cells 75 long intergenic noncoding RNAs (lincRNAs) were differentially expressed (38 up-regulated, 37 down-regulated) in response to IFN (S2 Fig), including NEAT1, a lincRNA that has previously been associated with viral infections [34–36]. As other genomes become increasingly well annotated, it will be possible to resolve a more in-depth understanding of the impact that ncRNAs play in the control of the innate immune system.

### Evolution of ISGs

**Fig 4:**
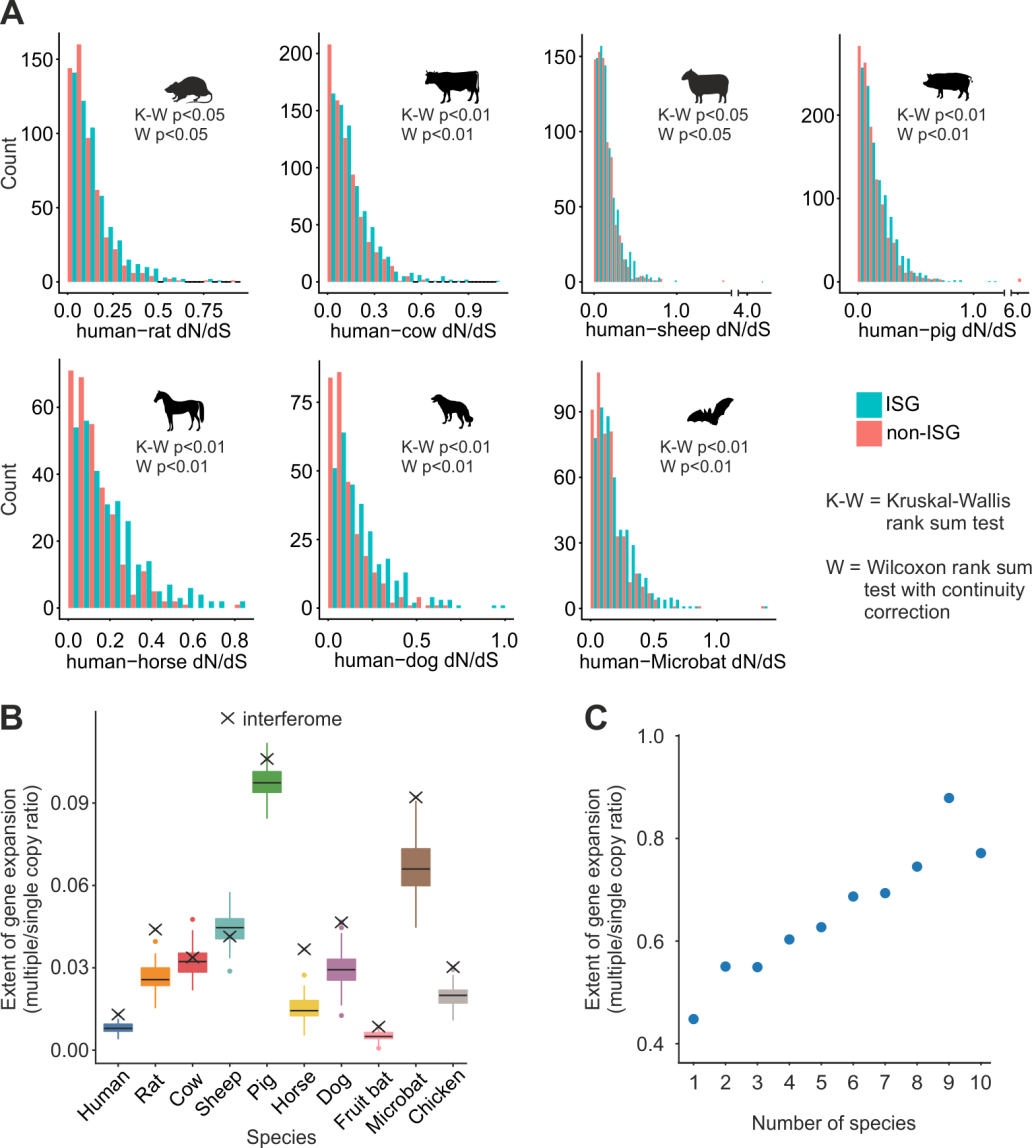
Evolutionary properties of ISGs. (A) For each nonhuman species, ISGs with one-to-one orthologs that were up-regulated in the human interferome and an identical number of random genes not differentially expressed by IFN stimulation were selected. dN and dS values were then retrieved from the Ensembl database. Histograms show dN/dS ratio values for ISGs (blue) and non-ISGs (red). Differences in the distribution of dN/dS values of the non-ISGs compared to ISGs were tested using the Kruskal–Wallis rank sum test and Wilcoxon rank sum test with continuity correction. (B) The extent of gene expansion was compared between ISGs and the genome as a whole. The y-axis represents the ratio between the number of genes for which there are paralogs (multiple) and those which are orthologs (single) as a proxy for gene expansion. Boxes and whiskers represent the values for 500 randomly selected non-ISGs, while ‘×’ represents the mean value for the ISGs for each species. With the exception of the sheep, all ISG values were above the median value. The code used for the random sampling and the generation of Fig 4A and 4B is provided in S3 Data and S4 Data, respectively, with the input file available as S5 Data. (C) Up- or down-regulated genes were divided into bins according to the number of species in which they were differentially expressed. The extent of gene expansion was calculated as panel B (S1 Data). A positive trend was identified for up-regulated genes whereby the greater the number of species which up-regulate a gene, the greater the likelihood of copies being retained (*P* < 0.05). IFN, interferon; ISGs, interferon-stimulated genes; K-W, Kruskal-Wallis; W, Wilcoxon rank sum test.

Virus–host coevolution has shaped the innate immune system, most frequently by placing antiviral genes under positive selection. We assessed the dN/dS ratios (as compared to the human) for one-to-one ISG orthologs. We observed that the overall distributions of dN/dS values of ISGs were significantly higher than those of randomly selected non-ISGs (Fig 4A). In addition, we assessed the copy number of each ISG across the different species studied here. Strikingly, for each species—with the exception of sheep and, to a lesser extent, cow—the proportion of ISGs with gene expansions was significantly above that of the genome as a whole (Fig 4B). Interestingly, sheep and cows are the only species with multiple copies of the IFN-β gene (generally a single copy gene in mammals).

The data described above suggest that, in general, expanded ISGs (compared to the rest of the genome) have an increased likelihood of conferring a selective advantage to the host species. Indeed, we observed that ISGs that are shared between multiple species have a higher likelihood of being expanded in the genome compared to other genes (*P* < 0.001, Fig 4C). Furthermore, among the core^vert^ ISGs, one-to-one orthologs are induced by IFN to a significantly higher level than genes present as paralogs (two-way ANOVA, F = 2.284, *P* < 0.05).

**Fig 5:**
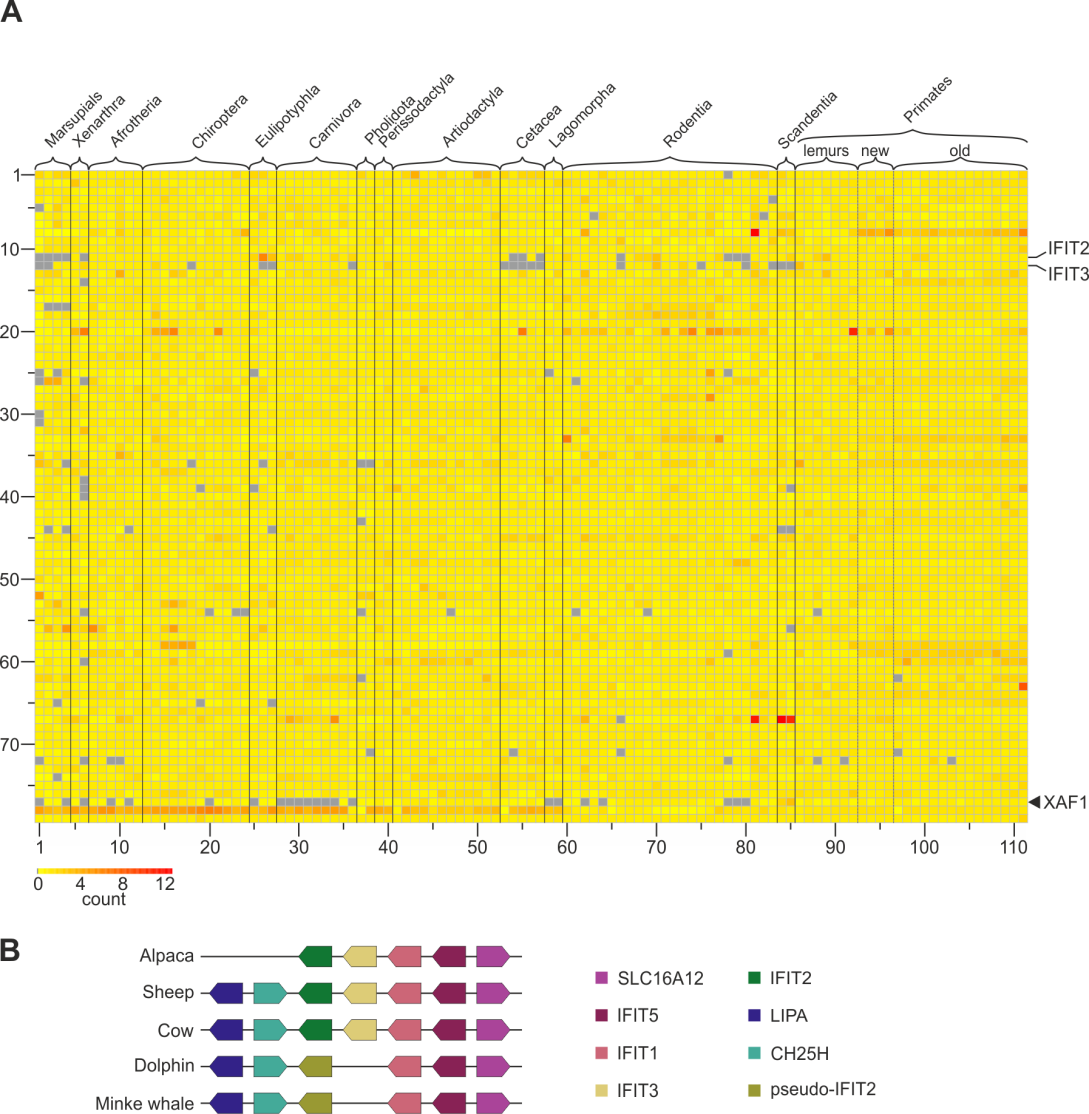
In silico screening of core^mamm^ ISGs in mammalian genomes. (A) A heatmap displaying the results of a similarity search–based screen of 111 mammalian genomes for sequences disclosing homology to mammalian core ISGs. Each column represents a distinct mammalian species, while each row represents a core^mamm^ ISG. Numbers on the left of each row identify each ISG as listed in S3 Table. Column numbers refer to species (S4 Table). Colours are proportional to the number of matches found in each genome, normalised by the median hit count for that gene across the Mammalia. Because the method is based upon sequence-similarity screening, high count levels for a particular gene do not necessarily reflect gene expansion. Note that the grey boxes indicate that no matches were identified, either due to a bona fide deletion or as a result of relatively poor quality of the genomes. Note that only 79 core^mamm^ ISGs are included in the analysis. Some of the core^mamm^ ISGs were excluded from this analysis because of their high levels of similarity between each other posing a risk for spurious results. (B) A cartoon of syntenic loci showing the absence of IFIT3 and pseudogenisation of IFIT2 in cetaceans. ISGs, interferon-stimulated genes.

We further analysed gene expansions and deletions among the core^mamm^ ISGs in the genomes of 111 mammalian species using an in silico sequence-similarity screening approach [37]. Although the uneven quality of the genomes used in the analysis make this approach prone to artefacts, we were able to detect core^mamm^ ISG deletions. For example, we observed that XAF1, which is a negative regulator of inhibitors of apoptosis, is deleted in cats (Felidae) (Fig 5A). In addition, we confirmed previously published deletions of IFIT3 among the Scandentia (tree shrews), Cetacea (whales and dolphins), and marsupials (Fig 5A) [38,39]. Furthermore, we observed that IFIT2 exists as a pseudogene in the Cetacea (Fig 5B).

## Discussion

In this study, we devised a systematic approach to unveil fundamental properties of the type I IFN system in vertebrates. We investigated the IFN response in several mammalian species and the chicken using a consistent experimental framework. By grouping ISGs and IRGs according to the number of species in which they were differentially expressed, we were able to reveal key facets related to the evolution and function of the IFN response.

We identified 62 core^vert^ ISGs that were up-regulated both in the chicken and nine mammalian species. Similarities between the chicken and mammalian IFN systems likely reflect fundamental functions that were present in the common ancestor of birds and mammals that have remained conserved over the ensuing circa 300 million years. Orchestration of the adaptive immune response by IFN appears to be a fully conserved and prioritised function among vertebrates. Specifically, within the core^vert^ ISGs, we found MHC class I components, along with genes involved at all levels of the antigen presentation process and genes involved in cell signalling to diverse cells of the adaptive immune system. Interestingly, we found that the type I IFN response may also facilitate local expression of factors of the complement system.

Only a limited set of genes relating to PAMP detection (MDA5, LGP2, TLR3) and downstream signalling (IRF1, IRF7, STAT1 and STAT2) were within the core^vert^ ISGs. In general, we noticed a greater number of IFN-up–regulated RNA sensors (and a greater level of their up-regulation) compared to DNA sensors. These data imply that the type I IFN response biases the sensitivity of surveillance for RNA viruses and may also reflect the difficulty, and danger, of differentiating self from nonself RNA in the cytoplasm.

Known antiviral ISGs are, in general, shared by many species. It therefore appears that a large proportion of the known antiviral capability of the IFN system evolved at an early point, a finding consistent with the presence of antiviral activity among fish ISGs [40,41].

Characterisation of the core^vert^ ISGs has also revealed several genes that hitherto have had little, if any, association in previous studies with the IFN response. Our data suggest that these genes play fundamental roles in the innate immune response of vertebrates that remain yet to be discovered. It is possible that genes such as these have been overlooked in previous studies simply as a result of their relatively modest fold up-regulation in response to IFN treatment in cells derived from individual animal species. This dataset therefore provides additional power with which to uncover novel genes central to the IFN system and an alternative approach by which to prioritise their relative biological significance and evolutionary conservation.

We observed that genes have arisen as ISGs throughout evolution, to the extent that certain genes are responsive to IFN only in particular phylogenetic groups. In addition, ISGs shared by multiple species have a higher propensity to be retained in genomes, yet another example of the pressure exerted by invading pathogens shaping vertebrate evolution. Hence, the result of these evolutionary processes is that every species possesses a unique repertoire of ISGs. These findings may help explain the differing sensitivities of certain animal species to specific viruses. For example, it has been widely hypothesised that the bat innate immune system has unique features that allow this species to withstand persistent, asymptomatic infection with viruses that are pathogenic in humans [42–44]. Our data reveal that the overall pattern of the bat interferome is relatively unremarkable: they up-regulate the core ISGs, have similar distributions of up-regulated and down-regulated genes, and up-regulate lineage (order Chiroptera), as well as species-specific, ISGs. However, we found that the basal transcription level of the type I interferome (including the known antiviral ISGs) to be higher in both the megachiropteran and microchiropteran cells compared to cells from other species (S5 Fig), a finding consistent with the previous observation that the interferon alpha (IFN-α) locus is constitutively active in bats [44]. Hence, bat cells might exist in a relatively constitutively active antiviral state.

The IRGs were differentially expressed to a conspicuously lower extent than ISGs, although the overall pattern was largely uniform across the species. The ability to assemble lists of shared IRGs allowed us to suggest that epigenetic control via down-regulation of genes involved in acetylation and methylation may be a relatively underappreciated function of the IFN response. For example, we found that ANP32A, a protein involved in acetyltransferase inhibition [30], was down-regulated by IFN in human, rat, sheep, cow, and pig cells. ANP32A was recently identified as a key cellular cofactor for avian influenza virus (AIV). Indeed, the avian influenza virus polymerase functions relatively poorly in mammalian cells, and this is due, at least in part, to the inability of AIV polymerase to bind efficiently to mammalian ANP32A [31]. It is intriguing that, in our data, chicken ANP32A is not significantly down-regulated by IFN.

Our study, like many of a similar nature, relies on the quality of the annotations of the genomes used. Indeed, many ISGs have been shown to have complex orthologies, and it is possible that some genes are misannotated or not yet annotated in the Ensembl Compara database. In order to decrease the impact of this factor in our data, we manually curated all ISGs that were initially identified in at least eight animal species. In addition, the use of primary cells for most species (in most cases derived from different individuals) reduced the possibility of artefacts deriving from cells that were passaged extensively in vitro. We also ensured that all RNAseq experiments were carried out in cells in which IFN stimulation resulted in an antiviral state (see Materials and methods). The system-level nature of RNAseq experiments and downstream bioinformatic analyses complicates direct comparisons between distinct studies. MORC3, for example, was previously suggested to be specifically up-regulated by the megabats, as it was not up-regulated in human A549 cells [45]. By contrast, in our study, we observed MORC3 among the core^vert^ genes, albeit robustly up-regulated in the fruit bat cells (> 12-fold) and minimally in human cells (< 2-fold). Similarly, the scope of this study is limited to type I IFN and a single cell type per species. It will be interesting in future studies to observe the differences in interferomes generated by IFN-γ and IFN-λ and, additionally, the variation that exists between cell types.

It is notable that the list of species for which we generated interferomes includes wild (rat, microbat, and fruit bat), as well as domestic companion (dog and horse) and livestock (pigs, chicken, cow, and sheep), species. We observed clear species- and lineage-specific ISGs for every species examined, which, as more genomes become sequenced, can be explored for evidence of how, for example, the domestication process has influenced the evolution of ISGs. Overall, the dataset described here represents the most comprehensive, cross-species ‘snapshot’ of the IFN response published to date. Our data provide a framework with which it will be possible to test hypotheses pertaining to the role of host innate immunity on virus emergence, cross-species transmission and pathogenesis.

## Materials and methods

### Ethics statement

Ex vivo tissue samples were collected postmortem either at commercial slaughterhouses or at the University of Glasgow School of Veterinary Medicine. In all cases, animals had been euthanized according to protocols approved by the local ethical committee and in accordance with the Council of the European Communities Directive of 24 November 1986 (86/609/EEC).

### Primary cell isolation and cell culture

Ex vivo skin samples were collected from chickens (*n* = 3), cows (*n* = 4), sheep (*n* = 3), horses (*n* = 3), a dog (*n* = 1), and pigs (*n* = 4) and primary fibroblasts isolated following an explant procedure. Briefly, the hair or feathers were removed from the skin prior to disinfection by soaking in 70% ethanol. After rinsing in PBS, the skin was removed from the underlying tissues, cut into circa 3-mm square explants, and added to cell culture dishes (without media, squamous surface uppermost) for one hour at 37°C before adding Dulbecco’s modified Eagle medium (DMEM) (Gibco) supplemented with 10% fetal bovine serum (FBS) (Gibco), 1% penicillin/streptomycin (p/s) (Sigma) and 100 U/ml nystatin (Sigma). Human primary dermal fibroblasts were purchased from PromoCell (catalogue number C-12302). Rat primary dermal fibroblasts were purchased from the European Collection of Authenticated Cell Cultures (ECACC) (catalogue number 06090769). *M*. *lucifugus* (little brown bat, representative of the microbats) primary dermal fibroblast cells, isolated from individuals caught in Oregon, United States of America, were kindly provided by William Kohler [46]. *P*. *vampyrus* (the large flying fox fruit bat, representative of the megabats) cells (PVK4) are an immortalised kidney cell line kindly provided by Megan Shaw [45]. The origin of each cell used in this study is summarized in S5 Table.

Human, rat, and dog cells were cultured in fibroblast growth medium 2 (PromoCell) supplemented with 10% FBS and p/s. 293T and PVK4 were all cultured in DMEM supplemented with 10% FBS and p/s. All cell cultures were incubated at 37°C with 5% CO2 in a humidified atmosphere.

### Virus infectivity assays

IFN- or mock-treated cells were challenged with infectious VLPs of envelope minus vesicular stomatitis virus (VSV-ΔG-GFP) decorated with a VSV-G envelope (provided in trans during VLP production) in order to confirm the antiviral state of the cells at the time of RNA harvest essentially as already described [47,48]. Briefly, cells were harvested by trypsinization and fixed in 5% formaldehyde. The number of infected cells in IFN- and mock-treated cells was assessed by flow cytometry.

### IFN treatment of cells

Parallel sets of cells were plated in multiwell plates 24 or 48 hours prior to IFN treatment and incubated at 37°C. Cells were treated with either 1,000 U/ml Universal interferon (UIFN, PBL InterferonSource), 200 ng/ml canine IFNα (Kingfisher), 1,000 U/ml porcine IFNα1 (Stratech), or 200 ng/ml chicken IFNα (AbD Serotec). Mock treatment was performed in parallel using DMEM lacking IFN. Cells were incubated for the indicated time period at 37°C, washed with PBS, and either lysed in Trizol (Thermo Fisher) for RNA extraction or challenged with VSV-ΔG-GFP to assess the antiviral state. Cells were only further processed for RNAseq analyses when they were in an antiviral state. In this study, cells were considered in an antiviral state when IFN stimulation induced at least 75% inhibition of VSV-ΔG-GFP infectivity (value chosen as average of three independent experiments) (S1 Fig). Pilot experiments were performed for each cell type in order to optimise conditions necessary for cells to reach an antiviral state. With the exception of dog cells, all cells reached an antiviral state after four hours of IFN treatment. The antiviral state in the primary canine cells isolated in these experiments required 24 hours of treatment with canine IFNα (S1 Fig). For comparative purposes, a complete set of experiments ranging from IFN stimulation to interferome analysis was performed in parallel in pig cells stimulated with either UIFN or porcine IFN-α (S3 Fig).

### RNA extraction and sequencing

RNA was extracted using Trizol (Thermo Fisher) and RNeasy (Qiagen) protocols. Briefly, chloroform was added to the RNA-containing phase of the Trizol sample and centrifuged. The aqueous phase was then mixed with ethanol and purified using RNeasy columns, incorporating an on-column DNase step (Qiagen) to ensure the complete removal of genomic DNA. Total RNA samples were quantified using the Qubit (Thermo Fisher) and were assessed for integrity using the Bioanalyser pico eukaryotic II RNA chip (Agilent). Only samples with an RNA integrity number (RIN) value > 9 were taken forward for library preparation.

Libraries of mock- and IFN-treated cell RNA were assembled using equal masses of total RNA. The External RNA Controls Consortium (ERCC) spike control was added to the total RNA sample in order to assess library quality following sequencing. RNA samples were first enriched for mRNA by selecting poly(A) RNA using the Dynabeads mRNA DIRECT Micro purification kit (Thermo Fisher). The eluted RNA was used to generate RNAseq libraries using the Ion Total RNA-seq Kit v2 (Thermo Fisher) following the manufacturer’s instructions, with the exception that RNA samples were sheared for just 1.5 minutes. Amplified and purified libraries were checked for quality and quantity using the Agilent Tapestation (D1000 tape) and Qubit (hsDNA assay). Libraries were run on the Ion Proton (Thermo Fisher) according to the manufacturer’s instructions.

### Data QC

Raw data were trimmed and assessed for quality using FASTQC (https://www.bioinformatics.babraham.ac.uk/projects/fastqc/). We performed multidimensional scaling (MDS) of normalised counts per million data using EdgeR (Bioconductor) in order to assess the impact of IFN and assessed biological covariance as a further control for data quality.

In order to assess the presence of cell culture contaminants on the transcriptomic data, we used Kraken to assign taxonomic labels to the reads using the MiniKraken database, which contains all complete bacterial, archaeal, and viral genomes in RefSeq [49]. To assess mycoplasma levels in the cell cultures, we used Bowtie2 to map the data to six different strains of mycoplasma known as frequent contaminants of cell cultures (S4 Fig).

### Data processing and differential expression analysis

Reads were aligned to host genomes (S6 Table) using a two-step procedure. A first round of mapping used TopHat2, followed by a second round of mapping using Bowtie2 in an attempt to map the remaining unmapped reads [50]. HTSeq-Count [51] was used to count reads mapping to genes annotated in.gtf files. Genes with <1 read mapping in at least half of the samples were removed prior to differential gene expression analysis using the EdgeR package (Bioconductor) [52,53]. FDR values were calculated using the Benjamini–Hochberg method. MDS and statistical analyses of the data were performed in R.

### Generation of orthologous gene clusters

We utilised the Ensembl Compara database [54], combined with our expression data, to generate a table of orthologs with the following associated data for each species: Ensembl ID, Gene name, log2FC, and FDR following IFN treatment. The Compara database provides a thorough account of gene orthology based upon whole genomes available in Ensembl and thus provided us with a standardised approach by which to define phylogenetically the clusters of orthologous genes relative to the chicken taken as an outgroup in the orthology assignment. However, certain gene families relating to innate immunity (for example, the IFITM genes) have undergone lineage-specific expansion, potentially resulting in genes not being annotated and clustered within the database [55]. To account for the misannotation and absence of genes in Compara, we improved the table by manually checking (and annotating, if necessary) genes initially found to be ‘missing’ in either one, two, or three of the 10 species analysed in this study. For this subset of genes, we searched for the presence of an as-yet-unannotated ortholog in the Ensembl genomes using blastn. In cases where a clear ortholog was detected, we included this gene within the appropriate orthogroup. In total, we identified an additional 18 genes that were added to the final table. In cases where a gene was not annotated in the Ensembl genome but a sequence (or predicted sequence) for the homologous species was available in NCBI, we mapped the RNAseq reads to the gene of the homologous species using Bowtie2. The number of reads mapping from each sample was then counted. In cases where an ortholog was present in NCBI from a closely related species, a relaxed Bowtie2 algorithm was first used to map reads to the sequence. The consensus sequence of the resulting contig was then used to count the reads in individual samples using Bowtie2 as above. Differential expression values were then determined using EdgeR having appended the results to the HTseq file. In total, 64 orthologs were added to the table as a result of the orthologous sequence mapping approach. Finally, we modified the .gtf file of sheep to reannotate STAT4 (to become STAT1 and STAT4) and SOCS1, and manually annotated the ZCCHC2 gene in the cow .gtf file.

### Verifying species-unique genes

Using the human genome as a gold standard for annotation, we next assessed the authenticity of each “species-specific” ISG (i.e., an ISG present only in one of the 10 species analysed in this study) based upon its current annotation within the genomes. We first generated a subset of species-specific genes by applying an arbitrary cutoff of their differential expression of ≥2 log2FC. This cutoff resulted in a total of 102 genes across eight species. A total of 33 genes (32%) were in this category as a result of misannotation. In particular, the current little brown bat and pig genomes appear to be currently less well annotated compared to other genomes, as a large proportion (73% and 79%, respectively) of seemingly species-unique genes were in reality misannotations.

### In silico genome screening

We used the database-integrated genome screening (DIGS) tool [37] to systematically screen the genomes of 111 mammalian species for sequences disclosing homology to 79 of the 90 core^mamm^ ISGs. The peptide sequences of the human copies of these 79 genes (obtained from BioMart [56]) were used as probes for tBLASTn-based searches of each species genome. Sequences disclosing above-threshold similarity to peptide probes were extracted and classified by BLASTx-based comparison to a reference library. This library contained, for each ISG, peptide sequences of human paralogs and orthologs from selected additional mammal species. The DIGS tool captures screening results in a relational database, wherein they could be interrogated using structured query language. The number of significant matches for each gene was determined using a gene-specific bitscore cutoff. These counts were normalised to the median value across the mammalian genomes screened to account for variation in exon numbers. Because DIGS is based upon sequence-similarity screening, high counts for a particular gene do not necessarily reflect bona fide gene expansion. Where no matches to a given gene were identified and no ortholog had been annotated in Ensembl, we attempted to confirm that deletion had occurred by viewing the corresponding genomic region in the UCSC and Genomicus genome browsers [57,58] and by comparing alignments of the orthologous genomic regions derived from species with and without the deleted gene. Deletions could not always be confirmed mainly due to low coverage or relatively poor quality assembly of the available genomes.

### dN/dS ratios for vertebrate interferomes

Clusters comprising one-to-one orthologs present in the interferome of each species were extracted and filtered to check for the presence of a gene in species X matching a human Ensembl ID. The human Ensembl ID was then used to query BioMart to extract the corresponding pairwise dN and dS values for species X against the human. Values for differential expression (log2FC) and FDR values for species X were merged with the dN/dS values and histograms of both significant (ISG) and nonsignificant (non-ISG) clusters plotted. dN and dS values were not available in Ensembl for the Large flying fox or the chicken. The overall distributions of dN/dS values for ISGs and non-ISGs were compared using the Kruskal–Wallis rank sum test and Wilcoxon rank sum test.

### Webserver

In order to allow mining of our data by the wider research community, we created a web-based public interactive data server, accessible at http://isg.data.cvr.ac.uk. The server hosts a database containing the orthologous ISG clusters studied in this paper. This web tool allows users to search for and download orthologous clusters and the associated experimental results. The search may be based on various search criteria:

1. Search clusters by HUGO Gene Nomenclature Committee (HGNC) gene name or Ensembl ID.
2. Select clusters based on the presence and expression characteristics in particular species.
3. Select clusters by presence or expression characteristics in a specific number of species.

It should be noted that genes can have different aliases to those in Ensembl, and these must be checked if a gene that is not initially present, e.g., MDA5, is present as IFIH1. These aliases are stated in Ensembl.

### URLs

The webserver for querying the dataset is available at http://isg.data.cvr.ac.uk/.

The DIGS blast framework is available at http://giffordlabcvr.github.io/DIGS-tool/.

### Accession numbers

The raw fastq files generated during this project have been submitted to the European Bioinformatics Institute (EBI) under project accession number PRJEB21332.

## Supporting information

S1 Fig Generation of transcriptomes used in this study. (A) Schematic outline of the experimental approach used to generate the interferomes used in this study. Parallel sets of cell cultures were used simultaneously. One set was used to determine the antiviral status of each cell line, while the other set was used to extract RNA and prepare libraries for sequencing. Cells were treated with either type I IFN or mock treated with cell culture media. Cells used for RNA extraction and library preparation were lysed in Trizol and stored at −80°C. The remaining samples were washed with PBS and challenged with VSV-ΔG-GFP. The infection status was assessed using flow cytometry. The extent of the antiviral state was determined by comparing infected (green) and uninfected (grey) cells using flow cytometry. (B) A graph showing the levels of infection by VSV-ΔG-GFP of mock-treated (grey bars) and IFN-treated (blue bars) cells. Results were normalised to the level of infection by VSV-ΔG-GFP of mock-treated cells (taken as 100%) and are available in S1 Data. GFP, green fluorescent protein; IFN, interferon; PBS, phosphate buffered saline; VSV, vesicular stomatitis virus.
(TIF)

S2 Fig ISG encoded transcript types. Barplots showing the types of transcripts generated by human genes significantly up-regulated (left panel) or down-regulated (right panel) in response to IFN. Transcript types were retrieved using BioMart from the Ensembl database. Some genes are annotated with > 1 transcript type; if one of the types was protein coding, this was assumed to be the canonical product. A total of 90% and 92% of up-regulated and down-regulated genes, respectively, are classified as protein coding genes. ncRNAs were present among both up-regulated and down-regulated genes (S1 Data). IFN, interferon; ISG, interferon-stimulated gene; lincRNA, long intergenic noncoding RNA; miRNA, microRNA; ncRNAs, noncoding RNAs.
(TIF)

S3 Fig A comparison of pig interferomes resulting from treatment with either UIFN or porcine IFNα. Interferomes were generated in primary pig fibroblasts using either porcine IFNα (*n* = 3) or UIFN (*n* = 3) (S1 Data). (A) A heatmap shows the log2FC values obtained for the core^vert^ ISGs in response to either UIFN or porcine IFNα. A high level of concordance was observed for the core genes in response to the different IFNs. Porcine IFNα up-regulated orthologs of every core gene with the exception of NLRC5. (B) Venn diagrams showing the overlap in the genes that are up-regulated (top) or down-regulated (bottom) by UIFN or porcine IFNα. A total of 89.1% of genes up-regulated by porcine IFNα were also up-regulated by UIFN. Similarly, 82.1% of genes down-regulated in response to porcine IFNα were also down-regulated by UIFN. (C) A scatterplot showing the log2FC in expression of ISGs up-regulated by either UIFN (X axis) or porcine IFNα (Y axis), where every gene is plotted as a point. The goodness of fit was assessed using the coefficient of determination, showing that genes were up-regulated to similar extents by both UIFN and porcine IFNα. IFN, interferon; log2FC, log2 fold change; UIFN, Universal interferon.
(TIF)

S4 Fig Reads generated for each dataset and mycoplasma screening. Mycoplasma can be detected in every deep sequencing experiment as either environmental or cell culture contaminants (S1 Data). Stacked barplots show the reads generated as part of this study where every bar represents a sample. Reads were assessed using FASTQC, trimmed, and mapped to the appropriate genome. Reads mapping to the host genome were then counted. Dark blue bars are mock-treated samples, and sky blue bars are those treated with IFN. Unmapped reads were further screened using genomes from six species of mycoplasma using Bowtie2 and the counts stacked on to the blue bars (coloured according to mycoplasma species). Only the fruit bat samples had appreciable levels of mycoplasma present.
(TIF)

S5 Fig Constitutive expression of ISGs in cells used in this study. Barplots showing the median basal FPKM levels for the whole interferome (left) or specific ISGs encoding known antiviral factors (right) for cells derived from each species analysed in this study in the absence of type I IFN treatment. Antiviral ISGs used in these graphs are provided in S2 Table. Both microbats and megabats show statistically higher basal FPKM levels compared to every non-Chiropteran species (Kruskal–Wallis, < 0.05). The data used to derive this figure are accessible in S1 Data. FPKM, fragments per kilobase mapped values; IFN, interferon; ISGs, interferon-stimulated genes.
(TIF)

S1 Table Number of differentially expressed orthologous clusters/genes in response to type I IFN. (DOCX)

S2 Table Antiviral genes analysed in this study. (DOCX)

S3 Table Core mammalian ISGs shown in Fig 5A. (DOCX)

S4 Table Species and genome versions used for the DIGS analysis. (DOCX)

S5 Table Origin of cells used in this study. (DOCX)

S6 Table Genome statistics. (DOCX)

S1 Data The raw values relative to the figures displayed in this study. This file contains the data used to plot Figures displayed in the manuscript. R code and separate files used as inputs for the R code are present as additional files.
(XLSX)

S2 Data R code used to generate Fig 3B. This R code requires Data S5 as an input, referred to in the code as Big_table_v3.6.1.
(TXT)

S3 Data R code used to generate Fig 4A. This R code requires Data S5 and Data S6 as input files, referred to in the code as Big_table_v3.6.1 and ASEPs, respectively.
(TXT)

S4 Data R code used to generate Fig 4B. This R code requires Data S5 as an input, referred to in the code as Big_table_v3.6.1.
(TXT)

S5 Data Input file for R code used to generate Figures Fig 3B, Fig 4A and Fig 4B. This file, referred to as Big_table_v3.6.1 in the R code, contains data required to run the code present in S2 Data, S3 Data and S4 Data that is used to generated Fig 3B, Fig 4A and Fig 4B, respectively.
(XLSX)

S6 Data List of confirmed antiviral genes required as input for S2 Data. This file contains data required to run the code present in S2 Data that is used to generate Fig 3B. This file is referred to as ASEPs in the R code.
(XLSX)

## Abbreviations

AIV: avian influenza virus
DIGS: database-integrated genome screening
DMEM: Dulbecco’s modified Eagle medium
EBI: European Bioinformatics Institute
ECACC: European Collection of Authenticated Cell Cultures
ERCC: External RNA Controls Consortium
FBS: fetal bovine serum
FDR: false discovery rate
FPKM: fragments per kilobase mapped values
HGNC: HUGO Gene Nomenclature Committee
IFN: interferon
IFN-α: interferon alpha
IFN-α: interferon beta
IFN-γ: interferon gamma
IFN-λ: interferon lambda
IRGs: interferon-repressed genes
ISGs: interferon-stimulated genes
lincRNAs: long intergenic noncoding RNAs
log2FC: log2 fold change
MDS: multidimensional scaling
MHC: major histocompatibility complex
ncRNAs: noncoding RNAs
PAMPs: pathogen-associated molecular patterns
p/s: penicillin/streptomycin
RIN: RNA integrity number
RNAseq: RNA sequencing
RSV: respiratory syncytial virus
SNP: single nucleotide polymorphism
UIFN: Universal interferon
