# Supplementary figures and images for: Fundamental properties of the mammalian innate immune system revealed by multispecies comparison of type I interferon responses
Source: PLoS Biol. 2017 Dec 18;15(12):e2004086. doi: 10.1371/journal.pbio.2004086 (PMC5747502; doi:10.1371/journal.pbio.2004086)

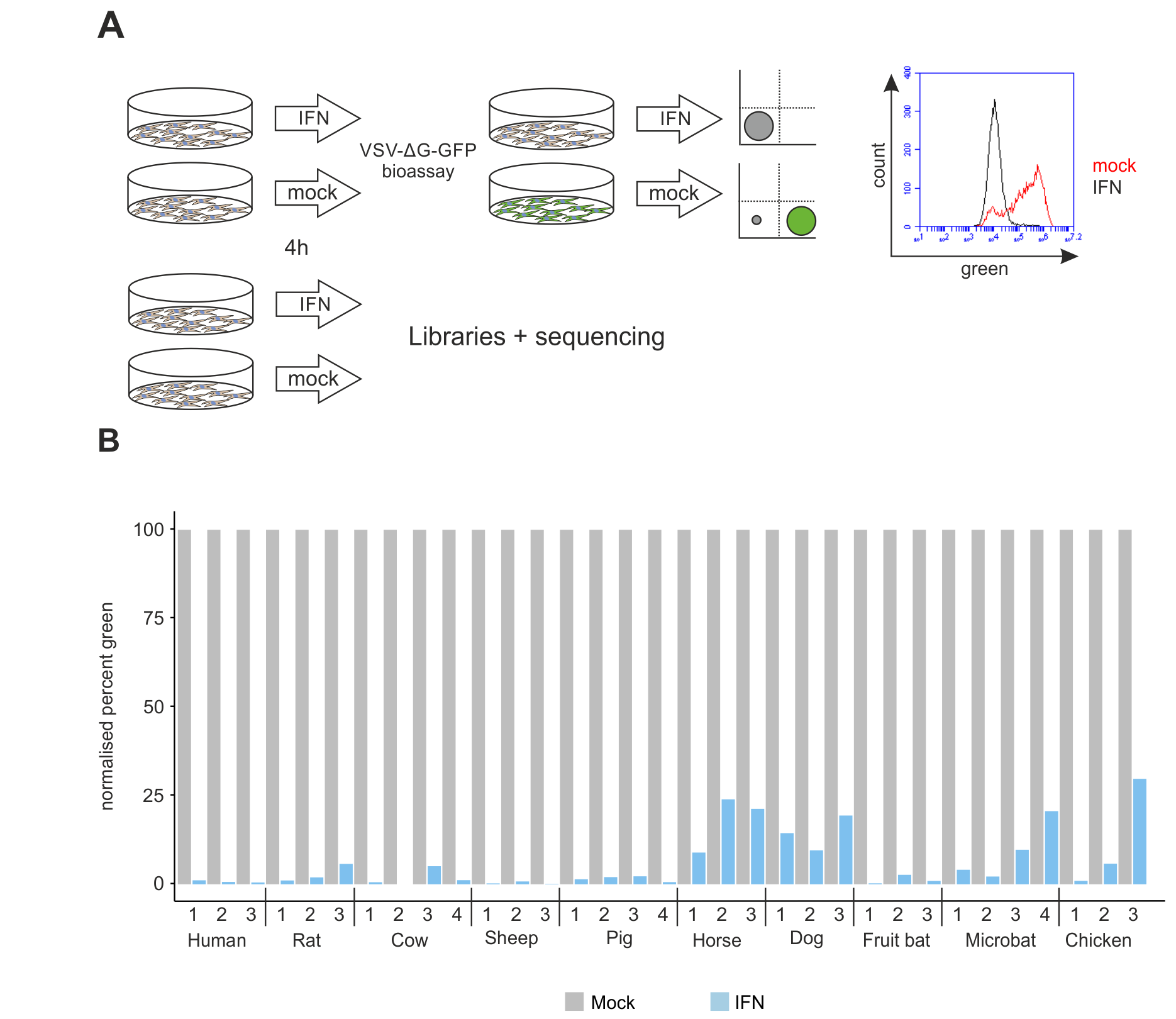

Supplement: S1 Fig — (A) Schematic outline of the experimental approach used to generate the interferomes used in this study. Parallel sets of cell cultures were used simultaneously. One set was used to determine the antiviral status of each cell line, while the other set was used to extract RNA and prepare libraries for sequencing. Cells were treated with either type I IFN or mock treated with cell culture media. Cells used for RNA extraction and library preparation were lysed in Trizol and stored at −80°C. The remaining samples were washed with PBS and challenged with VSV-ΔG-GFP. The infection status was assessed using flow cytometry. The extent of the antiviral state was determined by comparing infected (green) and uninfected (grey) cells using flow cytometry. (B) A graph showing the levels of infection by VSV-ΔG-GFP of mock-treated (grey bars) and IFN-treated (blue bars) cells. Results were normalised to the level of infection by VSV-ΔG-GFP of mock-treated cells (taken as 100%) and are available in S1 Data. GFP, green fluorescent protein; IFN, interferon; PBS, phosphate buffered saline; VSV, vesicular stomatitis virus. (TIF) [file pbio.2004086.s001.tif]

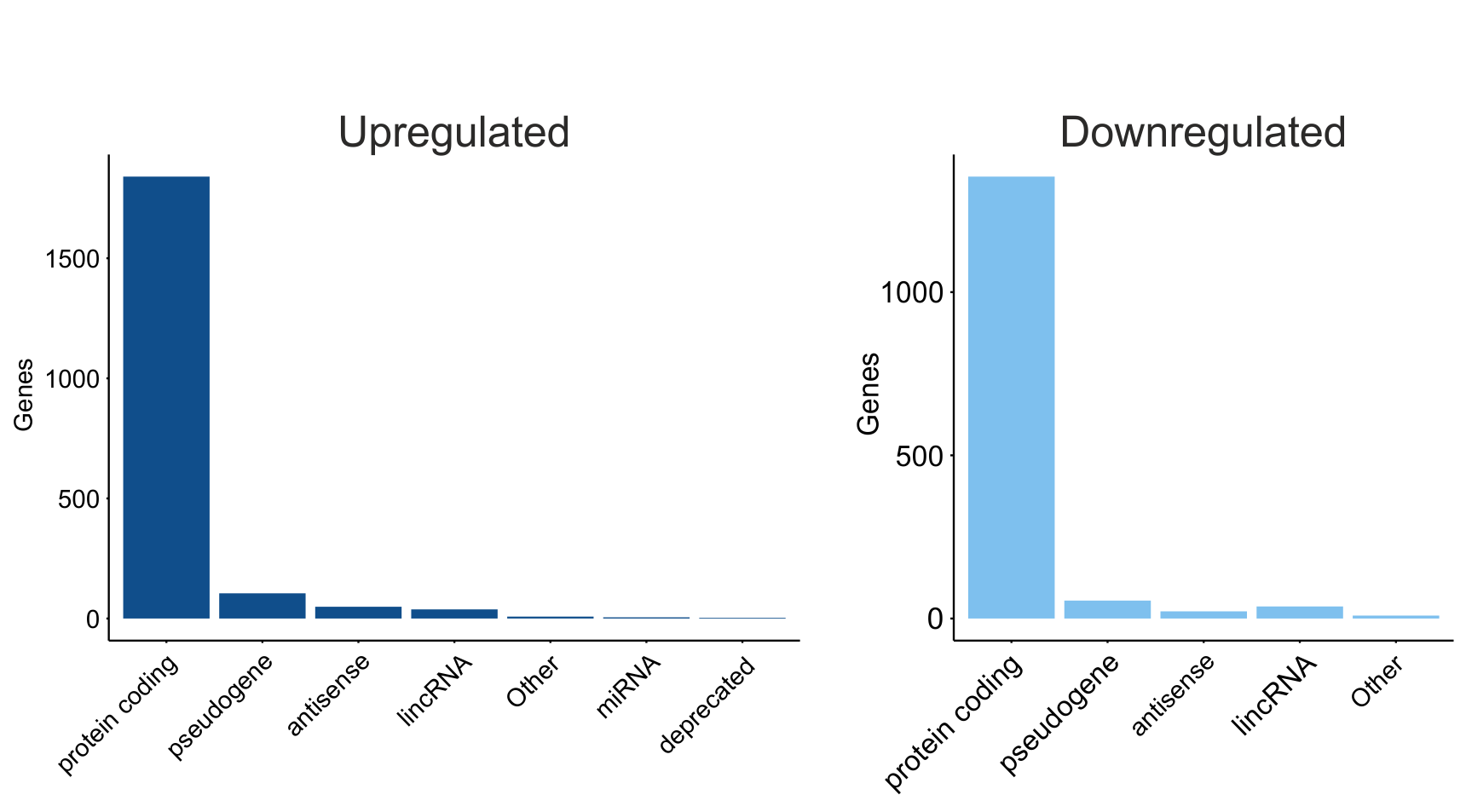

Supplement: S2 Fig — Barplots showing the types of transcripts generated by human genes significantly up-regulated (left panel) or down-regulated (right panel) in response to IFN. Transcript types were retrieved using BioMart from the Ensembl database. Some genes are annotated with > 1 transcript type; if one of the types was protein coding, this was assumed to be the canonical product. A total of 90% and 92% of up-regulated and down-regulated genes, respectively, are classified as protein coding genes. ncRNAs were present among both up-regulated and down-regulated genes (S1 Data). IFN, interferon; ISG, interferon-stimulated gene; lincRNA, long intergenic noncoding RNA; miRNA, microRNA; ncRNAs, noncoding RNAs. (TIF) [file pbio.2004086.s002.tif]

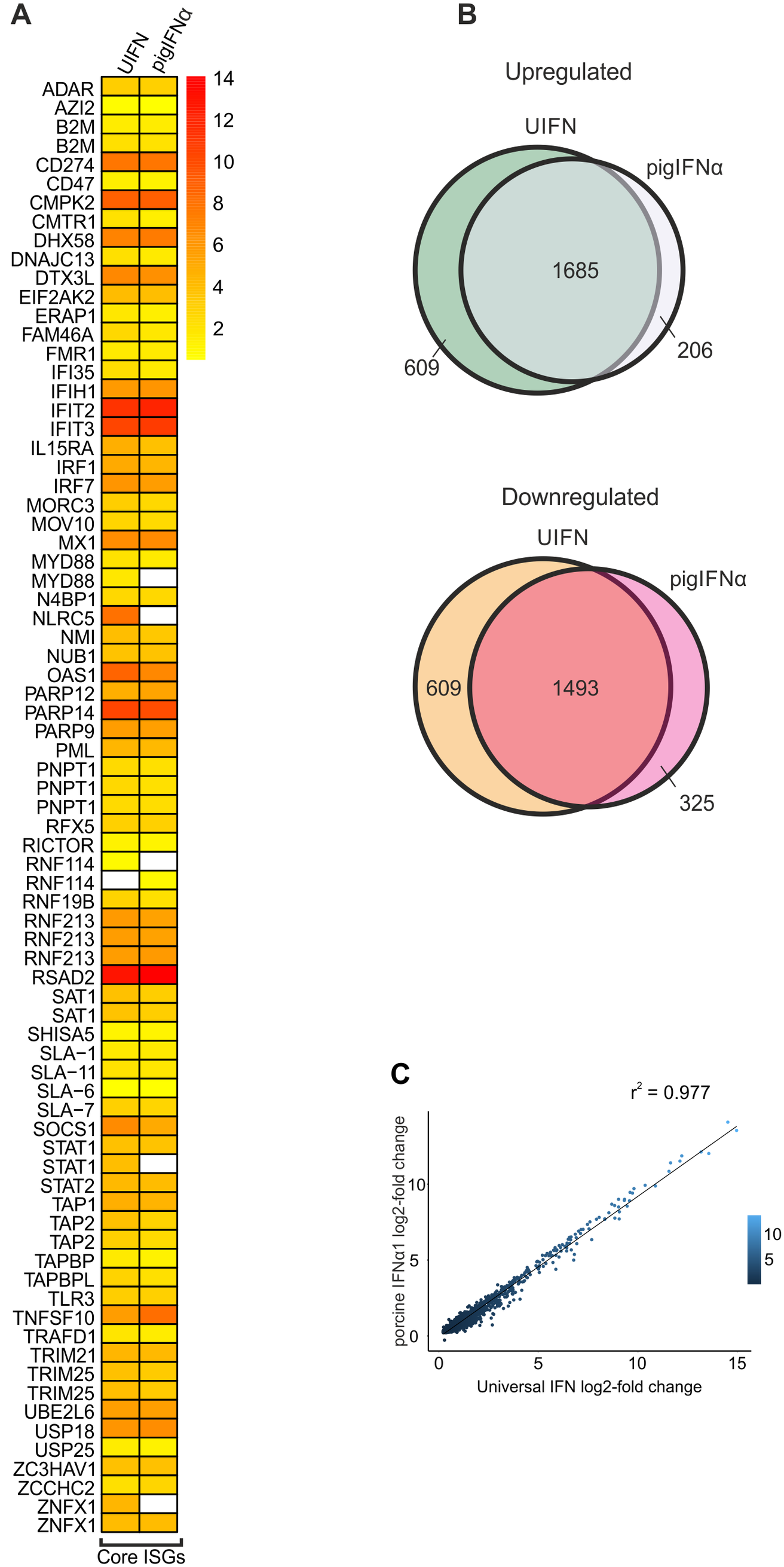

Supplement: S3 Fig — Interferomes were generated in primary pig fibroblasts using either porcine IFNα (n = 3) or UIFN (n = 3) (S1 Data). (A) A heatmap shows the log2FC values obtained for the corevert ISGs in response to either UIFN or porcine IFNα. A high level of concordance was observed for the core genes in response to the different IFNs. Porcine IFNα up-regulated orthologs of every core gene with the exception of NLRC5. (B) Venn diagrams showing the overlap in the genes that are up-regulated (top) or down-regulated (bottom) by UIFN or porcine IFNα. A total of 89.1% of genes up-regulated by porcine IFNα were also up-regulated by UIFN. Similarly, 82.1% of genes down-regulated in response to porcine IFNα were also down-regulated by UIFN. (C) A scatterplot showing the log2FC in expression of ISGs up-regulated by either UIFN (X axis) or porcine IFNα (Y axis), where every gene is plotted as a point. The goodness of fit was assessed using the coefficient of determination, showing that genes were up-regulated to similar extents by both UIFN and porcine IFNα. IFN, interferon; log2FC, log2 fold change; UIFN, Universal interferon. (TIF) [file pbio.2004086.s003.tif]

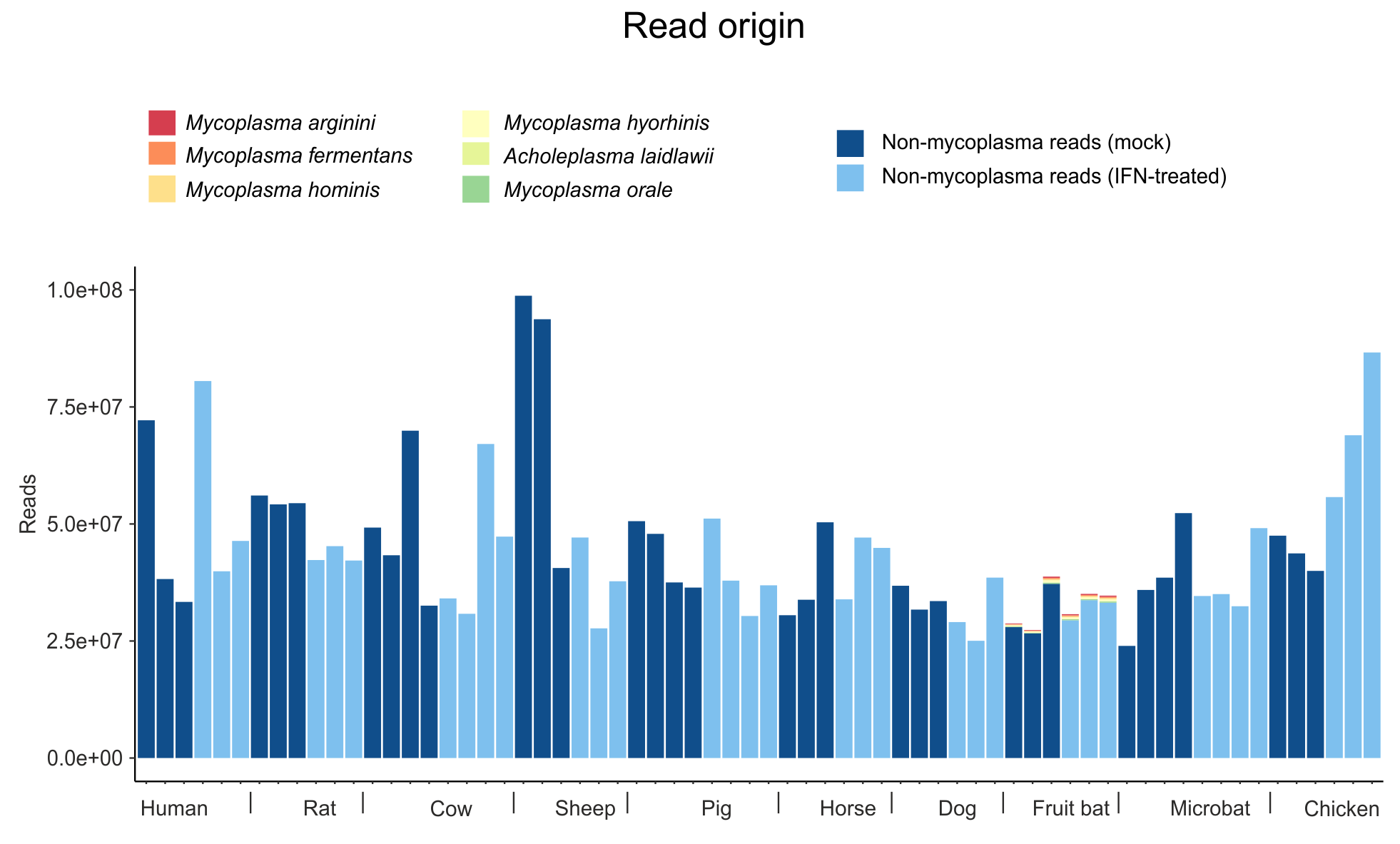

Supplement: S4 Fig — Mycoplasma can be detected in every deep sequencing experiment as either environmental or cell culture contaminants (S1 Data). Stacked barplots show the reads generated as part of this study where every bar represents a sample. Reads were assessed using FASTQC, trimmed, and mapped to the appropriate genome. Reads mapping to the host genome were then counted. Dark blue bars are mock-treated samples, and sky blue bars are those treated with IFN. Unmapped reads were further screened using genomes from six species of mycoplasma using Bowtie2 and the counts stacked on to the blue bars (coloured according to mycoplasma species). Only the fruit bat samples had appreciable levels of mycoplasma present. (TIF) [file pbio.2004086.s004.tif]

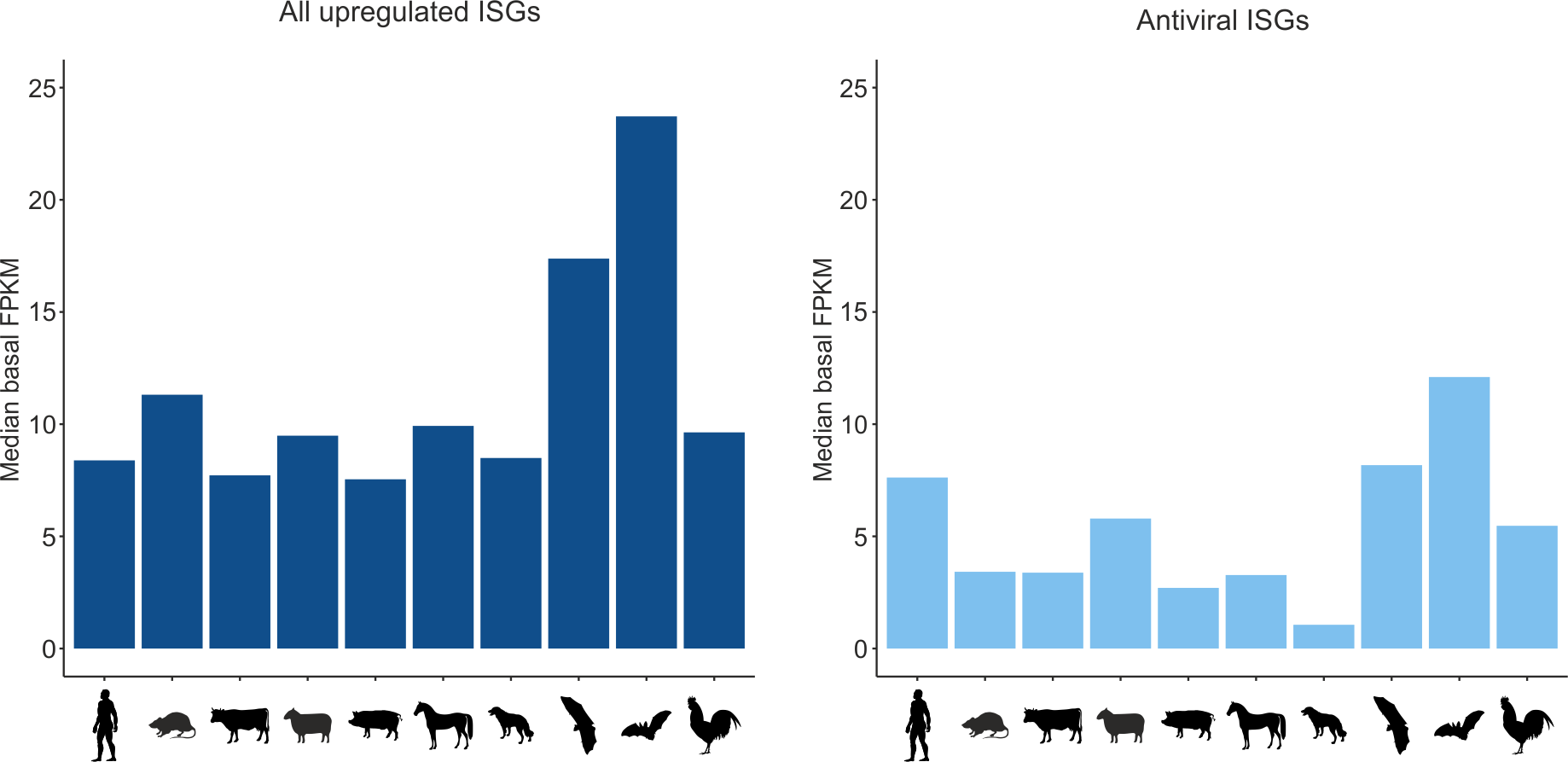

Supplement: S5 Fig — Barplots showing the median basal FPKM levels for the whole interferome (left) or specific ISGs encoding known antiviral factors (right) for cells derived from each species analysed in this study in the absence of type I IFN treatment. Antiviral ISGs used in these graphs are provided in S2 Table. Both microbats and megabats show statistically higher basal FPKM levels compared to every non-Chiropteran species (Kruskal–Wallis, < 0.05). The data used to derive this figure are accessible in S1 Data. FPKM, fragments per kilobase mapped values; IFN, interferon; ISGs, interferon-stimulated genes. (TIF) [file pbio.2004086.s005.tif]
