# Supplementary material for: Fundamental properties of the mammalian innate immune system revealed by multispecies comparison of type I interferon responses
Source: PLoS Biol. 2017 Dec 18;15(12):e2004086. doi: 10.1371/journal.pbio.2004086 (PMC5747502; doi:10.1371/journal.pbio.2004086)
Supplement: S1 Table — (DOCX) [file pbio.2004086.s006.docx]

**Table S1. Number of differentially expressed orthologous clusters/genes in response to type I IFN**

| **Species** | **ISGs** | **IRGs** |
| --- | --- | --- |
| Human | 2030* [2048]** | 1481 [1484] |
| Rat | 998 [1033] | 627 [626] |
| Cow | 1408 [1450] | 1030 [1036] |
| Sheep | 1358 [1391] | 988 [995] |
| Pig | 2096 [2247] | 2519 [2609] |
| Horse | 564 [598] | 195 [197] |
| Dog | 562 [575] | 389 [391] |
| Large flying fox (*P. vampyrus*) | 1414 [1428] | 1082 [1084] |
| Little brown bat (*M. lucifugus*) | 937 [1051] | 377 [379] |
| Chicken | 1124 [1054] | 670 [633] |

*numbers of orthologous clusters.

**number of genes.
