# Supplementary material for: Fundamental properties of the mammalian innate immune system revealed by multispecies comparison of type I interferon responses
Source: PLoS Biol. 2017 Dec 18;15(12):e2004086. doi: 10.1371/journal.pbio.2004086 (PMC5747502; doi:10.1371/journal.pbio.2004086)
Supplement: S2 Table — (DOCX) [file pbio.2004086.s007.docx]

**Table S2. Antiviral genes analysed in this study.**

| **Gene name** | **Human Ensembl ID** |  | **Gene name** | **Human Ensembl ID** |
| --- | --- | --- | --- | --- |
| ADAR | ENSG00000160710 |  | MOV10 | ENSG00000155363 |
| APOBEC3 (APOBEC3F) | ENSG00000128394 |  | MX1 | ENSG00000157601 |
| APOBEC3 (APOBEC3G) | ENSG00000239713 |  | MX2 | ENSG00000183486 |
| BST2 (tetherin) | ENSG00000130303 |  | OAS1/2/3 (OAS1) | ENSG00000089127 |
| CH25H | ENSG00000138135 |  | OAS1/2/3 (OAS2) | ENSG00000111335 |
| EIF2AK2 (PKR) | ENSG00000055332 |  | OASL | ENSG00000135114 |
| GBP1,GBP2 (GBP1) | ENSG00000117228 |  | PARP12 | ENSG00000059378 |
| GBP5 | ENSG00000154451 |  | PML (TRIM19) | ENSG00000140464 |
| HERC5 | ENSG00000138646 |  | RNAseL | ENSG00000135828 |
| IDO1 | ENSG00000131203 |  | RSAD2 (viperin) | ENSG00000134321 |
| IFI16 | ENSG00000163565 |  | SAMHD1 | ENSG00000101347 |
| IFIT1/2/3/5 (IFIT1) | ENSG00000185745 |  | SAT1 | ENSG00000130066 |
| IFIT1/2/3/5 (IFIT2) | ENSG00000119922 |  | SHISA5 (=SCOTIN) | ENSG00000164054 |
| IFIT1/2/3/5 (IFIT3) | ENSG00000119917 |  | SLFN11 | ENSG00000172716 |
| IFITM1/2/3 (IFITM1) | ENSG00000185885 |  | SMC5/6 (SMC6) | ENSG00000163029 |
| IFITM1/2/3 (IFITM2) | ENSG00000185201 |  | SP100 | ENSG00000067066 |
| IFITM1/2/3 (IFITM3) | ENSG00000142089 |  | TRIM21 | ENSG00000132109 |
| ISG15 | ENSG00000187608 |  | TRIM22 | ENSG00000132274 |
| ISG20 | ENSG00000172183 |  | TRIM5 | ENSG00000132256 |
| MORC3 | ENSG00000159256 |  | ZC3HAV1 (ZAP) | ENSG00000105939 |
