# Supplementary material for: Fundamental properties of the mammalian innate immune system revealed by multispecies comparison of type I interferon responses
Source: PLoS Biol. 2017 Dec 18;15(12):e2004086. doi: 10.1371/journal.pbio.2004086 (PMC5747502; doi:10.1371/journal.pbio.2004086)
Supplement: S3 Table — (DOCX) [file pbio.2004086.s008.docx]

**Table S3. Core mammalian ISGs shown in Fig. 5a**

| **Row** | **Gene** | **Row** | **Gene** |
| --- | --- | --- | --- |
| 1 | B2M | 41 | DHX58 |
| 2 | ERAP1 | 42 | IFIH1 |
| 3 | NLRC5 | 43 | MYD88 |
| 4 | PSMB8 | 44 | DDX58 |
| 5 | PSMB9 | 45 | RNF114 |
| 6 | PSMB10 | 46 | STAT1 |
| 7 | PSME1 | 47 | STAT2 |
| 8 | PSME2 | 48 | TRIM25 |
| 9 | RFX5 | 49 | USP25 |
| 10 | ADAR | 50 | CD274 |
| 11 | IFIT2 | 51 | IFI35 |
| 12 | IFIT3 | 52 | NMI |
| 13 | ISG15 | 53 | PARP14 |
| 14 | ISG20 | 54 | SOCS1 |
| 15 | MORC3 | 55 | TRAFD1 |
| 16 | MOV10 | 56 | TRIM21 |
| 17 | MX1 | 57 | USP18 |
| 18 | OAS | 58 | CD47 |
| 19 | PARP12 | 59 | IL15RA |
| 20 | EIF2AK2 | 60 | LGALS9 |
| 21 | PML | 61 | RICTOR |
| 22 | RSAD2 | 62 | TNFSF10 |
| 23 | SAT1 | 63 | C2 |
| 24 | SHISA5 | 64 | CASP8 |
| 25 | ZC3HAV1 | 65 | CMPK2 |
| 26 | DTX3L | 66 | CMTR1 |
| 27 | HERC6 | 67 | DNAJA1 |
| 28 | N4BP1 | 68 | DNAJC13 |
| 29 | NUB1 | 69 | EHD4 |
| 30 | PARP9 | 70 | FAM46A |
| 31 | RBCK1 | 71 | FMR1 |
| 32 | RNF19B | 72 | SERTAD1 |
| 33 | RNF213 | 73 | SLC25A28 |
| 34 | RNF31 | 74 | SP110 |
| 35 | UBA7 | 75 | TDRD7 |
| 36 | UBE2L6 | 76 | WARS |
| 37 | AZI2 | 77 | XAF1 |
| 38 | IRF1 | 78 | ZCCHC2 |
| 39 | IRF7 | 79 | ZNFX1 |
| 40 | IRF9 |  |  |
