# Supplementary material for: Fundamental properties of the mammalian innate immune system revealed by multispecies comparison of type I interferon responses
Source: PLoS Biol. 2017 Dec 18;15(12):e2004086. doi: 10.1371/journal.pbio.2004086 (PMC5747502; doi:10.1371/journal.pbio.2004086)
Supplement: S4 Table — (DOCX) [file pbio.2004086.s009.docx]

**Table S4: Species and genome versions used for the DIGS analysis**

| **Column** | **Species** | **Common name** | **Genome/assembly name** |
| --- | --- | --- | --- |
| 1 | *Ornithorhynchus anatinus* | Platypus | ornAna1 |
| 2 | *Sarcophilus harrisii* | Tasmanian devil | sarHar1 (Devil_ref v7.0) |
| 3 | *Macropus eugenii* | Tammar wallaby | macEug2 (Meug_1.1) |
| 4 | *Monodelphis domestica* | Gray short-tailed opossum | MonDom5 |
| 5 | *Dasypus novemcinctus* | Nine-banded armadillo | dasNov3 (Dasnov3.0) |
| 6 | *Choloepus hoffmanni* | Hoffmann's two-toed sloth | ChoHof1 |
| 7 | *Loxodonta africana* | African bush elephant | LoxAfr3 |
| 8 | *Trichechus manatus* | West Indian manatee | triMan1 (TriManLat1.0) |
| 9 | *Procavia capensis* | Rock hyrax | proCap1 |
| 10 | *Chrysochloris asiatica* | Cape golden mole | ChrAsi1.0 |
| 11 | *Echinops telfairi* | Lesser hedgehog tenrec | echTel2 |
| 12 | *Orycteropus afer* | Aardvark | OryAfe1.0 |
| 13 | *Miniopterus natalensis* | Natal long-fingered bat | Mnat.v1 |
| 14 | *Mus spretus* | Algerian mouse | SPRET_EiJ_v1 |
| 15 | *Myotis brandtii* | Brandt's bat | ASM41265v1 |
| 16 | *Myotis lucifugus* | Little brown bat | myoLuc2 |
| 17 | *Myotis davidii* | David's myotis | ASM32734v1 |
| 18 | *Eptesicus fuscus* | Big brown bat | EptFus1.0 |
| 19 | *Pteronotus parnellii* | Parnell's mustached bat | ASM46540v1 |
| 20 | *Eidolon helvum* | Straw-coloured fruit | ASM46528v1 |
| 21 | *Pteropus vampyrus* | Large flying fox | pteVam1 (Ptevap1.0) |
| 22 | *Pteropus alecto* | Black flying fox | ASM32557v1 |
| 23 | *Rhinolophus ferrumequinum* | Greater horseshoe bat | ASM46549v1 |
| 24 | *Megaderma lyra* | Greater false vampire bat | ASM46534v1 |
| 25 | *Erinaceus europaeus* | European hedgehog | EriEur2 |
| 26 | *Sorex araneus* | Common shrew | sorAra2 |
| 27 | *Condylura cristata* | Star-nosed mole | ConCri1.0 |
| 28 | *Leptonychotes weddellii* | Weddell seal | LepWed1.0 |
| 29 | *Odobenus rosmarus* | Walrus | Oros_1.0 |
| 30 | *Mustela putorius furo* | Ferret | musFur1(MusPutFur1.0) |
| 31 | *Panthera tigris altaica* | Siberian tiger | PanTig1.0 |
| 32 | *Acinonyx jubatus* | Cheetah | aciJub1 |
| 33 | *Felis catus* | Domestic cat | felCat5 (Felis_catus-6.2) |
| 34 | *Canis familiaris* | Dog | canFam3 (CanFam3.1) |
| 35 | *Ailuropoda melanoleuca* | Giant panda | ailMel1 |
| 36 | *Ursus maritimus* | Polar bear | UrsMar_1.0 |
| 37 | *Manis pentadactyla* | Chinese pangolin | GCA_000738955.1 |
| 38 | *Manis javanica* | Sunda pangolin | ManJav1.0 |
| 39 | *Ceratotherium simum* | White rhinoceros | cerSim1 (CerSimSim1.0) |
| 40 | *Equus caballus* | Horse | equCab2 |
| 41 | *Vicugna pacos* | Alpaca | vicPac2 |
| 42 | *Sus scrofa* | Pig | susScr3 (Sscrofa10.2) |
| 43 | *Giraffa Camelopardalis tippelskirchi* | Masai giraffe | ASM165123v1 |
| 44 | *Bubalus bubalis* | Water buffalo | ASM165123v1 |
| 45 | *Bison bison* | American bison | Bison_UMD1.0 |
| 46 | *Bos taurus* | Cow | bosTau7 (Btau_4.6.1) |
| 47 | *Pantholops hodgsonii* | Tibetan antelope | PHO1.0 |
| 48 | *Ovis aires* | Sheep | oviAri3 (Oar_v3.1) |
| 49 | *Capra aegagrus* | Wild goat | CapAeg_1.0 |
| 50 | *Capra hircus* | Domestic goat | CHIR_2.0 |
| 51 | *Okapia johnstoni* | Okapi | ASM166083v1 |
| 52 | *Capreolus capreolus* | European roe deer | kmer631 |
| 53 | *Orcinus orca* | Killer whale | ANOL02 (Oorc_1.1) |
| 54 | *Balaenoptera bonaerensis* | Antarctic minke whale | ASM97880v1 |
| 55 | *Balaenoptera acutorostrata* | Common minke whale | balAcu1 |
| 56 | *Lipotes vexillifer* | Baiji | Lipotes_vexillifer_v1 |
| 57 | *Tursiops truncatus* | Common bottlenose dolphin | turTru2 (Ttru_1.4) |
| 58 | *Ochotona princeps* | American pika | ochPri3 |
| 59 | *Oryctolagus cuniculus* | European rabbit | oryCun2 |
| 60 | *Ictidomys tridecemlineatus* | Thirteen-lined ground squirrel | spetri2 |
| 61 | *Fukomys damarensis* | Damaraland mole-rat | DMR_v1.0 |
| 62 | *Elephantulus edwardii* | Cape elephant shrew | EleEdw1.0 |
| 63 | *Mesocricetus auratus* | Golden hamster | MesAur1.0 |
| 64 | *Jaculus jaculus* | Lesser Egyptian jerboa | JacJac1.0 |
| 65 | *Nannospalax galili* | Northern Israeli blind subterranean mole rat | S.galili_v1.0 |
| 66 | *Phodopus sungorus* | Djungarian hamster | Psun0.5 |
| 67 | *Neotoma lepida* | Desert woodrat | ASM167557v1 |
| 68 | *Ellobius talpinus* | Northern mole vole | ETalpinus_0.1 |
| 69 | *Ellobius lutescens* | Transcaucasian mole vole | ASM168507v1 |
| 70 | *Cricetulus griseus* | Chinese hamster | criGri1 (C_griseus_v1.0) |
| 71 | *Mus castaneus* | Southeastern Asian house mouse | CAST_EiJ_v1 |
| 72 | *Mus pahari* | Gairdner's shrewmouse | PAHARI_EIJ_v1.1 |
| 73 | *Mus caroli* | Ryukyu mouse | CAROLI_EIJ_v1.1 |
| 74 | *Mus musculus* | House mouse | mm10 (GRCm38) |
| 75 | *Apodemus sylvaticus* | Wood mouse | GCA_001305905.1 (ASM130590v1) |
| 76 | *Rattus norvegicus* | Brown rat | rn6 (Rnor_6.0) |
| 77 | *Marmota marmot marmota* | Alpine marmot | marMar2.1 |
| 78 | *Dipodomys ordii* | Ord's kangaroo rat | dipOrd1 (Dipord1.0) |
| 79 | *Cavia porcellus* | Guinea pig | cavPor3 |
| 80 | *Cavia aperea* | Brazilian guinea pig | CavAp1.0 |
| 81 | *Octodon degus* | Degu | OctDeg1.0 |
| 82 | *Heterocephalus glaber* | Naked mole-rat | hetGla2 (HetGla_female_1.0) |
| 83 | *Chinchilla lanigera* | Long-tailed chinchilla | ChiLan1.0 |
| 84 | *Tupaia chinensis* | Chinese tree shrew | TupChi_1.0 |
| 85 | *Tupaia belangeri* | Northern treeshrew | tupBel1 |
| 86 | *Galeopterus variegatus* | Sunda flying lemur | G_variegatus_3.0.2 |
| 87 | *Propithecus coquereli* | Coquerel's sifaka | Pcoq_1.0 |
| 88 | *Daubentonia madagascariensis* | Aye-aye | DauMad_1.0 |
| 89 | *Microcebus murinus* | Gray mouse lemur | micMur1 (ASM16544v1) |
| 90 | *Eulemur macaco* | Black lemur | Emacaco_refEf_BWA_oneround |
| 91 | *Eulemur flavifrons* | Blue-eyed black lemur | Eflavifronsk33QCA |
| 92 | *Otolemur garnettii* | Northern greater galago | otoGar3 |
| 93 | *Saimiri boliviensis* | Black-capped squirrel monkey | saiBol1 |
| 94 | *Aotus nancymaae* | Nancy Ma's night monkey | Anan_1.0 |
| 95 | *Cebus imitator* | White-headed capuchin | Cebus_imitator-1.0 |
| 96 | *Callithrix jacchus* | Common marmoset | calJac3 (Callithrix jacchus-3.2) |
| 97 | *Rhinopithecus bieti* | Black snub-nosed monkey | ASM169854v1 |
| 98 | *Rhinopithecus roxellana* | Golden snub-nosed monkey | Rrox_v1 |
| 99 | *Nasalis larvatus* | Proboscis monkey | nasLar1 |
| 100 | *Colobus angolensis palliatus* | Angolan colobus | Cang.pa_1.0 |
| 101 | *Cercocebus atys* | Sooty mangabey | Caty_1.0 |
| 102 | *Chlorocebus sabaeus* | Green monkey | ChlSab1.1 |
| 103 | *Papio anubis* | Olive baboon | papAnu2 (Panu_2.0) |
| 104 | *Mandrillus leucophaeus* | Drill | Mleu.le_1.0 |
| 105 | *Macaca nemestrina* | Southern pig-tailed macaque | Mnem_1.0 |
| 106 | *Macaca mulatta* | Rhesus macaque | rheMac3 (CR_1.0) |
| 107 | *Nomascus leucogenys* | Northern white-cheeked gibbon | Nleu1.0 |
| 108 | *Pongo abelii* | Orangutan | ponAbe2 (P_pygmaeus_2.0.2) |
| 109 | *Gorilla gorilla* | Gorilla | gorGor3 |
| 110 | *Pan troglodytes* | Chimpanzee | panTro4(Pan_troglodytes-2.1.4) |
| 111 | *Homo sapiens* | Human | hg38 (GRCh38) |
