# Supplementary material for: Fundamental properties of the mammalian innate immune system revealed by multispecies comparison of type I interferon responses
Source: PLoS Biol. 2017 Dec 18;15(12):e2004086. doi: 10.1371/journal.pbio.2004086 (PMC5747502; doi:10.1371/journal.pbio.2004086)
Supplement: S5 Table — (DOCX) [file pbio.2004086.s010.docx]

**Table S5. Origin of cells used in this study**

| **Species** | **Sample type** | **Replicate type** | **Tissue** |
| --- | --- | --- | --- |
| Human | Primary fibroblast cells | 3 x cell culture | Skin |
| Rat | Primary fibroblast cells | 3 x cell culture | Skin |
| Cow | Primary fibroblast cells | 4 independent animals | Skin |
| Sheep | Primary fibroblast cells | 3 independent animals | Skin |
| Pig | Primary fibroblast cells | 4 independent animals | Skin |
| Horse | Primary fibroblast cells | 3 independent animals | Skin |
| Dog | Primary fibroblast cells | 3 x cell culture | Skin |
| Large flying fox | Established cell line* | 3 x cell culture | Kidney |
| Microbat (*M. lucifugus*) | Primary fibroblast cells | 4 independent animals | Skin |
| Chicken | Primary fibroblast cells | 3 independent animals | Skin |
| Pig | Primary fibroblast cells | 4 independent animals | Skin |

*Glennon *et al*. (2015)[40].
