# Supplementary material for: Fundamental properties of the mammalian innate immune system revealed by multispecies comparison of type I interferon responses
Source: PLoS Biol. 2017 Dec 18;15(12):e2004086. doi: 10.1371/journal.pbio.2004086 (PMC5747502; doi:10.1371/journal.pbio.2004086)
Supplement: S6 Table — (DOCX) [file pbio.2004086.s011.docx]

**Table S6. Genome statistics**

| **Species** | **Reference genome** | **GTF version/date used** | **Protein coding genes** | **Pseudogenes** |
| --- | --- | --- | --- | --- |
| Human | GRCh37 | 2014 | 20,441 | 14,606 |
| Rat | Rnor_6.0 | 2015-6 | 22,263 | 1582 |
| Cow | UMD3.1 | 2011-9 | 19,994 | 797 |
| Sheep | Ovine3.1 | 2013-12 | 20,921 | 290 |
| Pig | Sscrofa10.2 | 2014-02 | 21,630 | 568 |
| Horse | EquCab2 | 2012-11 | 20,449 | 4400 |
| Dog | CanFam3.1 | 2015-7 | 19,856 | 950 |
| Fruit bat | pteVam1 | 2010-5 | 16,990 | 1033 |
| Microbat | Myoluc2.0 | 2011-6 | 19,728 | 4408 |
| Chicken | Galgal4 | 2013-12 | 15,508 | 42 |
